# Interactions between the gut microbiome and host gene regulation in cystic fibrosis

**DOI:** 10.1186/s13073-020-0710-2

**Published:** 2020-01-28

**Authors:** Gargi Dayama, Sambhawa Priya, David E. Niccum, Alexander Khoruts, Ran Blekhman

**Affiliations:** 1 Department of Genetics, Cell Biology and Development, University of Minnesota, Minneapolis, MN USA; 2 Department of Medicine, University of Minnesota, Minneapolis, MN USA; 3 Center for Immunology, BioTechnology Institute, University of Minnesota, Minneapolis, MN USA; 4 Department of Ecology, Evolution, and Behavior, University of Minnesota, Minneapolis, MN USA

**Keywords:** Cystic fibrosis, Host-microbe interactions, Gene regulation, Microbiome, Colorectal cancer

## Abstract

**Background:**

Cystic fibrosis is the most common autosomal recessive genetic disease in Caucasians. It is caused by mutations in the *CFTR* gene, leading to poor hydration of mucus and impairment of the respiratory, digestive, and reproductive organ functions. Advancements in medical care have led to markedly increased longevity of patients with cystic fibrosis, but new complications have emerged, such as early onset of colorectal cancer. Although the pathogenesis of colorectal cancer in cystic fibrosis remains unclear, altered host-microbe interactions might play a critical role. To investigate this, we characterized changes in the microbiome and host gene expression in the colonic mucosa of cystic fibrosis patients relative to healthy controls, and identified host gene-microbiome interactions in the colon of cystic fibrosis patients.

**Methods:**

We performed RNA-seq on colonic mucosa samples from cystic fibrosis patients and healthy controls to determine differentially expressed host genes. We also performed 16S rRNA sequencing to characterize the colonic mucosal microbiome and identify gut microbes that are differentially abundant between patients and healthy controls. Lastly, we modeled associations between relative abundances of specific bacterial taxa in the gut mucosa and host gene expression.

**Results:**

We find that 1543 genes, including *CFTR*, show differential expression in the colon of cystic fibrosis patients compared to healthy controls. These genes are enriched with functions related to gastrointestinal and colorectal cancer, such as metastasis of colorectal cancer, tumor suppression, p53, and mTOR signaling pathways. In addition, patients with cystic fibrosis show decreased gut microbial diversity, decreased abundance of butyrate producing bacteria, such as Ruminococcaceae and *Butyricimonas*, and increased abundance of other taxa, such as Actinobacteria and *Clostridium*. An integrative analysis identified colorectal cancer-related genes, including *LCN2* and *DUOX2*, for which gene expression is correlated with the abundance of colorectal cancer-associated bacteria, such as Ruminococcaceae and *Veillonella*.

**Conclusions:**

In addition to characterizing host gene expression and mucosal microbiome in cystic fibrosis patients, our study explored the potential role of host-microbe interactions in the etiology of colorectal cancer in cystic fibrosis. Our results provide biomarkers that may potentially serve as targets for stratifying risk of colorectal cancer in patients with cystic fibrosis.

**Electronic supplementary material:**

The online version of this article (10.1186/s13073-020-0710-2) contains supplementary material, which is available to authorized users.

## Background

Cystic fibrosis (CF) is the most common autosomal recessive genetic disease in Caucasians, where it occurs with a frequency of 1 in 3000 births [1]. CF is caused by mutations in the cystic fibrosis transmembrane conductor regulatory (*CFTR*) gene, which plays critical functions in epithelial ion transport and hydration of mucus. Absent or reduced *CFTR* activity results in thick, viscous secretions that impair functions of the respiratory, digestive, and reproductive organ systems.

Multiple advances in medical care in CF, once a fatal pediatric disease, have led to remarkable gains in patient life expectancy. However, increased longevity of CF patients into adulthood has led to new challenges, such as gastrointestinal cancer. The average onset of colorectal cancer (CRC) in CF patients is approximately 20–30 years earlier than in the general population [2, 3]. Systematic data on colonoscopic screening and surveillance suggest that CF-associated CRC arises via the classical adenoma to cancer sequence, but adenomatous polyps develop at a younger age in CF and progress faster to more advanced neoplasms [4]. In fact, loss of *CFTR* expression in tumors of non-CF patients has been associated with a worse prognosis in early-stage CRC [5]. Recently, specific recommendations for CRC screening were introduced in standard care of adult CF patients, which include earlier initiation of screening and shorter intervals for surveillance [6].

Although previous studies have identified *CFTR* as a tumor suppressor gene that may play a role in early onset of colon cancer [5, 7], the pathogenesis of CRC in CF remains unclear. A number of factors can be considered. It is likely that the altered microbiota composition and microbiota-mucosal interface are also the reasons for a chronic state of low-grade mucosal inflammation in CF [8]. Notably, *CFTR* is hyper-expressed in the stem cell compartment of the intestinal crypt [9], which is the site of CRC origination [10].

Than and colleagues have shown altered expression of genes involved in immune cell homeostasis and inflammation, mucins, cell signaling and growth regulation, detoxification and stress response, lipid metabolism, and stem cell regulation in the intestines of *CFTR* mutant mice [5]. The intestinal microbiota of these animals is also distinguished by lower bacterial community richness, evenness, and diversity, consistent with a major impact of *CFTR* deficiency on gastrointestinal physiology [11]. Altered fecal microbiome has also been demonstrated in a number of clinical CF cohorts, where it was characterized by decreased microbial diversity, lower temporal microbial community stability, and decreased relative abundances of taxa associated with health, such as *Faecalibacterium*, *Roseburia*, *Bifidobacterium*, *Akkermansia*, and *Clostridium cluster XIVa* [12–17]. Greater degrees of dysbiosis were noted to correlate with severity of CF disease phenotype, burden of antibiotics, and evidence for intestinal inflammation in diverse pediatric cohorts with varying degree of fat malabsorption.

Here, we compare the mucosal microbiome (via 16S rRNA sequencing) and colonic gene expression (via RNA-seq) in adult patients with CF and healthy controls undergoing CRC screening by colonoscopy. By using an integrative analysis approach, we identified correlations between host colonic gene expression and mucosal microbiome data. This allowed us to characterize potential interactions between host genes and microbes, providing insight on the early development of CRC in CF patients. We also hope these host gene-microbiome associations can serve as a precursor for designing future hypothesis-driven studies that can help tease out the directionality of causation.

## Methods

### Patients and mucosal biopsy samples

Mucosal biopsies were obtained from patients undergoing CRC screening and surveillance colonoscopies at the University of Minnesota (Additional file 1). The majority of CF patients receiving care at the Minnesota Cystic Fibrosis Center participate in a systematic colonoscopic CRC screening program as described previously [4]. None of the CF patients had acute infections within the preceding 3 months of the procedure, and CF patient colonoscopies were done for colon cancer screening and not acute gastrointestinal symptoms. Control samples were obtained from non-CF patients with average risk of CRC undergoing routine colonoscopic CRC screening or surveillance. Pinch biopsies, four per patient, were obtained using the Radial Jaw 4 Jumbo w/Needle 240 (length) forceps for a 3.2-mm working channel (Boston Scientific, Marlborough, MA; Catalog # M00513371) in the right colon and placed into RNAlater stabilization solution (Thermo Fisher Scientific, Waltham, MA). The protocol was approved by the University of Minnesota Institutional Review Board (IRB protocol 1408 M52889). Gene expression was analyzed by RNA-seq from a total of 33 samples obtained from 18 CF patients and 15 non-CF control participants (Additional file 2: Figure S1).

### RNA extraction and sequencing

Biopsy tissue was kept in the RNAlater stabilization solution overnight at 4 °C. RNA was prepared following tissue homogenization and lysis using the TRIzol Plus RNA Purification Kit (Thermo Fisher Scientific; catalog # 2183–555) following detailed manufacturer’s instructions. Total RNA samples were converted to Illumina sequencing libraries using Illumina’s Truseq Stranded mRNA Sample Preparation Kit (Cat. # RS-122-2103). Total RNA was oligo-dT purified using oligo-dT-coated magnetic beads, fragmented, and then reverse transcribed into cDNA. The cDNA was adenylated and then ligated to dual-indexed (barcoded) adaptors and amplified using 15 cycles of PCR. Final library size distribution was validated using capillary electrophoresis and quantified using fluorimetry (PicoGreen). Indexed libraries were then normalized, pooled, and then size selected to 320 bp ± 5% using Caliper’s XT instrument. Truseq libraries are hybridized to a paired-end flow cell, and individual fragments were clonally amplified by bridge amplification on the Illumina cBot. Once clustering is complete, the flow cell is loaded on the HiSeq 2500 and sequenced using Illumina’s SBS chemistry (Additional file 2: Figure S1).

### Host RNA-seq quality control, read mapping, and filtering

We performed quality check on raw sequences from all 33 samples (to assure better downstream analysis using FastQC) [18]. This helped assess any biases due to parameters such as quality of the reads, GC content, number of reads, read length, and species to which the majority of the reads mapped (Additional file 2: Figure S2). The FASTQ files for forward and reverse (R1 and R2) reads were mapped to the reference genome using kallisto [19], where an index for the transcriptomes was generated to quantify estimated read counts and TPM values. Mean distribution for the TPM values was plotted using R to filter all the transcripts below a threshold value of log2[TPM] < 0. We generated PCA plots using sleuth [20] to examine sample clusters and visualization of expression patterns for genes using bar plots (Additional file 2: Figures S3 and S4). For further analysis of outlier samples, box plots were generated using Cook’s distance and heat map clustered by condition and mutation status was generated for the top 20 expressed genes (Additional file 2: Figures S5 and S6).

### Host RNA-seq differential expression and enrichment analysis

To determine differentially expressed genes between CF and healthy samples, we quantified and annotated the transcripts using DESeq2 [21]. The output from kallisto was imported into DESeq2 using the tximport package [22]. The transcripts were annotated against the ensemble database using bioMART to obtain gene symbols [23]. Transcripts below a threshold of row-sum of 1 were filtered and collapsed at a gene symbol level. Prior to differentially expressed gene analysis, the read counts were normalized and the gene-wise estimates were shrunken towards the fitted estimates represented by the red line in the dispersion plot (Additional file 2: Figure S7). The gene-wise estimates that are outliers are not shrunk and are flagged by the blue circles in the plot (Additional file 2: Figure S7). DESeq2 applies the Wald’s test on estimated counts and uses a negative binomial generalized linear model determines differentially expressed genes and the log-fold changes (Additional file 2: Figure S8). The log-fold change shrinkage (*lcfshrink()*) function was applied for ranking the genes and data visualization. For data smoothing, MA plots were generated before and after log2 fold shrinkage. We found no change in the MA plot (Additional file 2: Figure S9) post smoothing, as there are no large log-fold changes in the current data (log2 fold change between − 1 and 1) due to low counts. The data were further transformed, and the normalized values were extracted using regularized logarithm (rlog) to remove the dependence of variance on mean. We used the Benjamini-Hochberg method for reducing the false discovery rate (FDR) with a cutoff of 0.05 for identifying differentially expressed genes for further analysis. Enrichment analysis was done using Ingenuity Pathway Analysis (IPA, QIAGEN Inc., https://www.qiagenbioinformatics.com/products/ingenuitypathway-analysis). The log-fold changes, *p* values, and FDR values (for all the genes with FDR < 0.05) were fed into IPA for both up- and downregulated differentially expressed genes between CF and healthy samples. Disease/functional pathways and gene networks were determined based on the gene enrichment. Furthermore, we looked at how many target upstream regulators were enriched based on our list of differentially expressed genes using IPA. We found 134 targets that passed the filter (*p* value < 0.01) from a total of 492 targets, of which 96 were transcription regulators.

### 16S rRNA extraction and sequencing

Mucosal biopsies samples (~ 3 × 3 mm) from 13 CF and 12 healthy individuals were collected in 1 mL of RNAlater and stored for 24 h at 4 °C prior to freezing at − 80 °C. DNA was extracted using a MoBio PowerSoil DNA isolation kit according to the manufacturer’s instructions (QIAGEN, Carlsbad, USA). To look at the tissue-associated microbiome, the V5-V6 region of 16S rRNA gene was amplified as described by Huse et al. [24] using the following indexing primers (V5F_Nextera: TCGTCGGCAGCGTCAGATGTGTATAAGAGACAGRGG ATTAGATACCC, V6R_Nextera: GTCTCGTGGGCTCGGAGATGTGTATAAGAGACAGCGACRRCCATGCANCACCT). Index and flowcell adaptors were added with this step. Forward indexing primer used is - **AATGATACGGCGACCACCGA**GATCTACAC [i5] TCGTCGGCAGCGTC and reverse indexing primers used is - **CAAGCAGAAGACGGCATACGA**GAT [i7]GTCTCGTGGGCTCGG. Post two rounds of PCR, pooled, size-selected samples were denatured with NaOH, diluted to 8 pM in Illumina’s HT1 buffer, spiked with 15% PhiX, and heat denatured at 96 °C for 2 min immediately prior to loading. A MiSeq 600 cycle v3 kit was used to sequence the sample.

### Gut mucosal microbiome data processing, quality assessment, and diversity analysis

We processed the FASTQ files using FastQC [18] to perform quality control on the raw sequences. We then used SHI7 [25] for trimming Nextera adaptors, stitching paired-end reads and performing quality trimming at both ends of the stitched reads until a minimum Phred score of 32 was reached. Following quality control, we obtained an average of 217,500 high-quality reads per sample (median 244,000; range 9551–373,900) with an average length of 281.9 bases and an average quality score of 37.19. These merged and filtered reads were used for closed reference operational taxonomic unit (OTU) picking and taxonomy assignment against GreenGenes database with 97% similarity level using the NINJA-OPS program [26].

To identify any potential contaminants originating from laboratory kits and reagents, we used two negative controls consisting of “blank” DNA extractions that were processed and sequenced alongside the true samples. The principal coordinates analysis (PCoA) plot of the true samples with the negative controls shows clustering by sample type (Additional file 2: Figure S10) suggesting that most sequences observed in true samples were not derived from reagent contamination. We used these sequenced negative controls for identification of contaminants by applying decontam, an R package that implements a statistical classification procedure to detect contaminants in 16S and metagenomic sequencing data and has been shown to identify contaminants across diverse studies, including those from biopsy samples [27]. We used the prevalence-based contamination identification approach that is recommended for low biomass environments, like tissue biopsy. This method computes a prevalence-based score (ranging from 0 to 1) that is used by decontam to distinguish between contaminant and non-contaminants. A small score (less than 0.5) indicates that a sequence feature is likely to be a contaminant, while higher score (greater than 0.5) indicates non-contaminants (i.e., true sequences). We plotted the distribution of prevalence-based scores assigned by decontam (Additional file 2: Figure S11) that shows that most of the OTUs in our samples were assigned high scores (> 0.5), thus suggesting non-contaminant origin. Nevertheless, in order to identify any potential contaminants, we ran decontam analysis at the default classification threshold of 0.1, and at a higher threshold of 0.2.

We performed alpha- and beta-diversity analysis in R using the vegan [28] and phyloseq [29] packages. We used resampling-based computation of alpha diversity, where the OTU table is subsampled 100 times at minimum read depth (9551 reads) across all samples and computed average richness estimate for each alpha-diversity metric (chao1, observed OTUs, and Shannon). Wilcoxon rank-sum test was used for testing the statistical significance of the associations between alpha diversity of the CF and healthy conditions. For computing beta-diversity, we first rarefied the OTU table (using vegan’s *rrarefy()* function) at a minimum sequence depth (i.e., 9551 reads) across the samples and then computed Bray-Curtis dissimilarity, weighted UniFrac, and unweighted UniFrac metrics. The Adonis test was used for assessing if there is significant association between the beta-diversity of the CF/healthy condition and the diversity results are plotted using the ggplot2 package in R.

### Gut mucosal microbiome differential abundance and functional analysis

We performed differential abundance testing between CF and healthy conditions using the phyloseq [29] package in R. We first created a phyloseq object from the OTU table (using the *phyloseq()* function) and filtered this object to only include OTUs occurring in at least half of the number of samples in the condition with fewer samples (i.e., min (number of samples in CF, number of samples in Healthy)/2)) with at least 0.1% relative abundance (using the *filter_taxa()* function). The filtered phyloseq object was converted into a DESeqDataSet object (using *phyloseq_to_deseq2()*), and the *DESeq()* function was invoked. This performed dispersion estimations and Wald’s test for identifying differentially abundant OTUs, with their corresponding log-fold change, *p* value, and FDR-adjusted *q* values between the CF and healthy conditions. We agglomerated the OTUs at different taxonomic ranks (using the *tax_glom()* function) and repeated the above steps to identify differentially abundant taxa at genus, family, order, class, and phylum levels.

We also tested for associations between taxonomic abundance and mutation status of CF samples. We first categorized samples into three genotype categories: (1) Healthy: Samples with no mutations; (2) CF_df508: CF samples with homozygous delta-F508 deletion, which is associated with more severe CF condition [30]; and (3) CF_other: CF samples with df508 heterozygous deletion or other mutation status. We used DESeq2’s likelihood ratio test (LRT) to identify taxa that showed significant difference in abundance across the three categories.

We then generated the predicted functional profiles for the gut microbes using PICRUSt v1.0.0 pipeline, [31] where pathways and enzymes are assigned using the Kyoto Encyclopedia of Genes and Genomes (KEGG) database. The KEGG level 3 pathways were filtered for rare pathways by only including pathways with relative abundance > 0.1% in at least half of the samples, normalized to relative abundance, and tested for association with CF/healthy conditions using non-parametric Wilcoxon rank-sum test followed by FDR adjustment.

To verify that our results were not affected by potential contaminants, we applied the prevalence-based contamination identification approach implemented in the decontam R package described above. We repeated the differential abundance analysis after removal of OTUs identified as contaminants and found the same microbes to be differentially abundant between CF and healthy samples or mutation status as those in the analysis without contamination identification. This confirmed that our results were not influenced by potential contaminants.

### Integrated analysis of interactions between host gene dysregulations and changes in microbiome

For this analysis, differentially expressed genes from host and gut microbial OTUs from their respective overlapping samples were used (22 samples in total, with 12 healthy samples and 10 CF samples). We further subset differentially expressed genes between CF and healthy conditions (FDR < 0.05), specifically enriched for gastrointestinal cancer disease pathways (524 genes). Using absolute expression log ratio greater than 0.35, we obtained a representative set of both up- and downregulated genes from these pathways, leaving 250 genes for downstream analysis. The OTU table was collapsed at the genus level (or the last characterized level) and filtered for rare taxa by only including taxa with at least 0.1% relative abundance present in at least half of the number of samples in the condition with fewer samples (i.e., min (number of samples in CF, number of samples in Healthy)/2)), resulting in 35 taxa for further processing. Following this, centered log ratio transform was applied on the filtered table. We then performed correlation analysis between host gene expression data for 250 genes and gut microbiome abundance data for 35 taxa (genus level) defined above. Spearman correlation was used for this analysis as it performs better with normalized counts (gene expression) as well as compositional data (microbiome relative abundance) compared to other metrics, such as Pearson correlation [32]. We computed the Spearman rank correlation coefficients and the corresponding *p* values using the *cor.test()* function with two-sided alternative hypothesis. A total of 8750 (250 genes × 35 taxa) statistical tests were performed, and *p* values were corrected for multiple comparisons using the qvalue package in R [33]. Representative gene-taxa correlations were visualized using corrplots [34] in R, where the strength of the correlation is indicated by the color and size of the visualization element (square) and the significance of the correlation is indicated via asterisk. We also computed the Sparse Correlation for Compositional Data (SparCC) [35] for the taxa found significantly correlated (*q* value < 0.1) with the CRC genes. Pseudo *p* values were computed using 100 randomized sets. Significant gene-microbe correlations (*q* value < 0.1) and significant microbe-microbe correlations (SparCC |R| > =0.1 and *p* value < 0.05) were visualized as a network using Cytoscape v3.5.1 [36].

To ensure that these correlations were not influenced by any potential contaminants, we repeated the analysis after removing any contaminants identified by decontam as described above and found that the associations remained unchanged. Additionally, we also verified whether any correlated taxa coincided with known lab contaminants mentioned by Salter and colleagues [37]. We found no overlapping microbes with the list of known contaminants, except *Pseudomonas*. *Pseudomonas* was not identified as a contaminant in our decontam analysis. Interestingly, *Pseudomonas aeruginosa*, which is a major pathogen in cystic fibrosis lung infection [38, 39], has previously been isolated from the fecal samples of patients with cystic fibrosis [17, 40]. This suggests that the presence of *Pseudomonas* in our samples is not due to contamination and could be potentially attributed to the cystic fibrosis condition of our patient cohort.

## Results

### Host RNA-seq sample preprocessing and quality assessment

We first examined gene expression in colonic biopsies from 18 CF and 15 healthy individuals. Overall, CF and healthy samples had comparable number of reads (28,250,473 and 30,041,827 reads on average, respectively) with the average quality greater than 30 phred score across all samples (Additional file 2: Figure S2). The sequences were annotated to generate estimated read counts and transcripts per kilobase million (TPM) using kallisto [19], resulting in 173,259 total transcripts, of which 56,283 passed the filter of mean TPM greater than 1 (TPM > 1). While the principal component analysis (PCA) plots showed an overlap between the expression profile of most samples from CF and healthy individuals, it identified two possible outliers (samples 1096 and 1117) (Additional file 2: Figure S3). In addition, the top five transcripts driving the PC were of mitochondrial origin (Additional file 2: Figure S4). Hence, to reduce any bias in identifying differentially expressed genes, we filtered out all the mitochondrial transcripts from the data. We further investigated the outliers using the remaining transcripts by calculating Cook’s distance between the samples and found that the two samples (1096 and 1117) were still outliers (Additional file 2: Figure S5). This was further evident by the heatmap of the top 20 most highly expressed genes (Additional file 2: Figure S6), where we found an alternate expression pattern for the two samples, compared to the rest. Therefore, the two outlier CF samples (1096 and 1117) were eliminated from further analysis.

### Differentially expressed host genes between CF and healthy mucosal samples

To examine gene expression differences we used read counts from the remaining 16 CF and 15 healthy samples. Using DESeq2, we identified 1543 differentially expressed genes at *q* value < 0.05 (Benjamini-Hochberg correction; see Additional file 2: Figure S8 for a volcano plot). Of the 1543 differentially expressed genes, 919 (59%) were upregulated and 624 (41%) were downregulated in CF patients. Including sex as a covariate in the model did not substantially alter the results (only 43 additional differentially expressed genes were identified); therefore, we did not include sex in downstream analyses. The full list of differentially expressed genes significant at *q* value < 0.05 is available in Additional file 3.

**Fig. 1:**
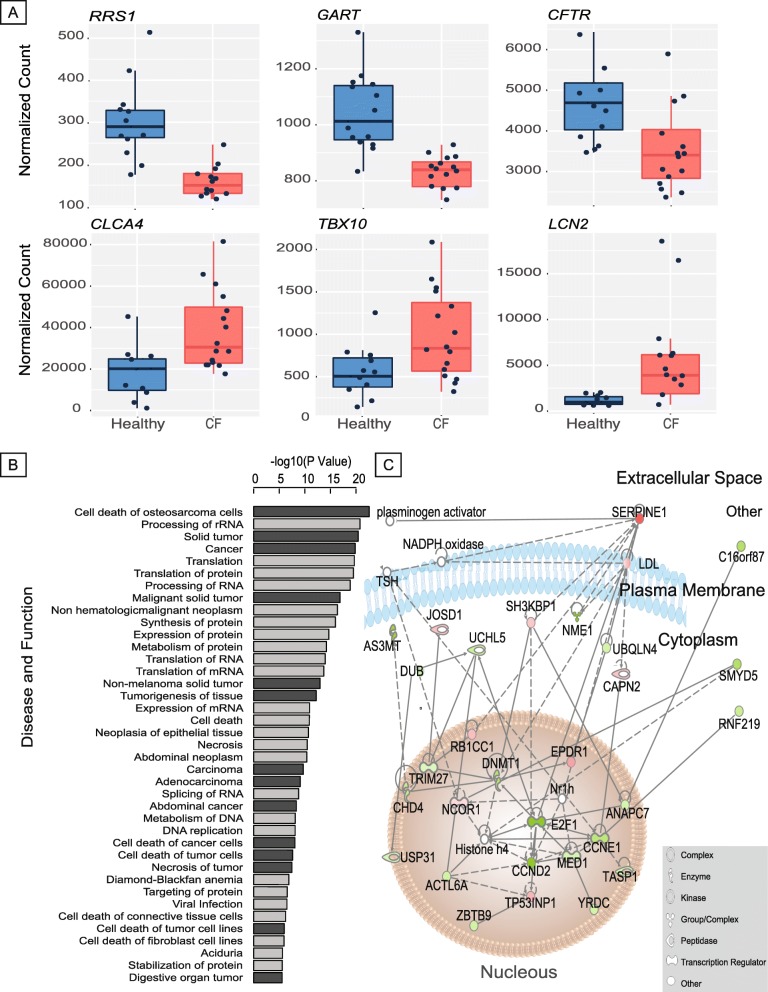
Differentially expressed (DE) genes in the host. **a** Box plot of six genes that are a part of the gastrointestinal cancer pathway (one of the key disease pathways influenced by DE gene at *q* value < 0.05 cutoff), showing differential expression between healthy and CF samples. **b** Disease and functional pathways that are most significantly enriched with DE genes (*q* value < 0.05), sorted by the *p* value (cut off − log10(*p* value) < 5). The dark gray bars represent cancer-related pathways. **c** Gastrointestinal cancer pathway gene network with upregulated genes represented in green and downregulated genes represented in red. The intensity of the color is indicative of higher (brighter) or lower (duller) difference in expression. The shapes represent each protein’s role (see legend) and the figure also illustrates the part of the cell they are most active in

We visualized the expression pattern of five (three upregulated and two downregulated) randomly selected differentially expressed representative genes and *CFTR*, from genes included in the colorectal cancer disease pathway (Fig. 1a). Consistent with the expectation of changes in mucosal immunity that could compensate for a diminished protective mucus function, we noted *LCN2* to be one of the top differentially expressed genes (*q* value = 2.54E−08, Wald’s test). *LCN2* encodes for lipocalin 2, which limits bacterial growth by sequestering iron-laden bacterial siderophore [41]. However, a number of other top genes are involved in major cellular biology processes and were previously related to cancer pathogenesis and colon cancer. Examples include *RRS1* (*q* value = 6.16E−09), which encodes for the ribosomal biogenesis protein homolog that promotes angiogenesis and cellular proliferation, but suppresses apoptosis [42]; *KRTAP5-5* (*q* value = 4.89E−08), which encodes for keratin-associated protein 5-5, a protein that plays important roles in cytoskeletal function and facilitates various malignant behaviors that include cellular motility and vascular invasion [43]; and *ALDOB* (*q* value = 2.64E−07), which encodes for aldolase B, an enzyme that promotes metastatic cancer-associated metabolic reprogramming [44]. Additional examples of differentially expressed genes (log-fold change > 0.5 and *q* value < 0.05), such as *CDH3*, *TP53INP2*, *E2F1*, *CCND2*, and *SERPINE1*, were also previously shown to have direct roles in colorectal and digestive cancers [45–47]. While some of these genes participate in basic cancer-related cellular functions such as proliferation and invasion [45, 47–50], others, e.g., *BEST2*, play important roles in gut barrier function and anion transport [51]. To test signatures of inflammation in our data, we intersected our DEGs (*q* value < 0.05) with data from Hong et al. [52], who compared gene regulation in Crohn’s disease (CD) patients (with and without inflammation) and healthy controls. Of the 43 genes enriched in CD patients with inflammation in their study [52], we only found 2 genes, *SERPINE1* and *APOB* that overlapped with our DEGs (Fisher’s exact test, *p* value = 1). In addition to the genes visualized in Fig. 1a, additional randomly selected differentially expressed genes are visualized in Additional file 2: Figure S12), showing expression pattern differences between the CF and healthy samples.

We next performed an enrichment analysis to categorize functional and disease pathways among differentially expressed genes (*q* value < 0.05) in IPA. The top canonical pathways (Additional file 2: Figure S13) are mostly responsible for signaling and regulatory functions, such as *EIF2* signaling (*p* value = 3.32E−35), mTOR signaling (*p* value = 3.83E−08) and regulation of chromosomal replication (*p* value = 1.60E−06). Of the 39 significantly enriched disease and functional pathways (*p* value < 1.00E−05; Fig. 1b), 14 are related to cancer, including gastrointestinal cancer (*p* value = 2.61E−06), abdominal cancer (*p* value = 9.23E−03), large intestine cancer (*p* value = 7.00E−05), and colorectal cancer (*p* value = 8.63E−03). In addition, using the list of differentially expressed genes, we found that the promoter sequences are enriched with binding sites of 96 potential transcription regulators (*p* value < 0.01; see “Methods”). Among these transcription factors, many have been previously shown to control cancer-related pathways. For example, *MYCN* and *KRAS* are prominently involved in neuroblastoma and colorectal cancer, respectively [53, 54]. *NHF4A* is involved in transcriptional regulation of many aspects of epithelial cell morphogenesis and function, which has been linked to colorectal cancer [55]. *CST5*, which encodes cytostatin D, is a direct target of p53 and vitamin D receptor and promotes mesenchymal-epithelial transition to suppress tumor progression and metastasis [56]. *E2F3* is a potent regulator of the cell cycle and apoptosis that is commonly deregulated in oncogenesis [57].

A metabolic network for the gastrointestinal (GI) cancer-related differentially expressed genes is shown in Fig. 1c, illustrating the interactions between genes that are upregulated in CF (e.g., *TP53INP1*, *SERPINE1*, *NCOR1*, and *CAPN2*) and downregulated in CF (*E2F1*, *MED1*, *ECND2*, and *AS3MT*), highlighting the cellular location of these genes’ product. Additional gene network for colorectal cancer can be found in Additional file 2: Figure S14), where the genes are also positioned in the region of the cell where they are most active. We found that genes such as *BEST2* (involved in ion transport) and *RUVBL1* (involved in cell cycle, cell division, and cell damage) are downregulated, while genes such as *TP53INP2* (involved in transcription regulation) and *CDH3* (involved in sensory transduction) are upregulated. Given the predicted role of gene regulation in colorectal cancer and the dysregulation of CRC-related pathways, these results may help understand mechanisms controlling early onset of colon cancer in cystic fibrosis.

### Difference in microbiome composition between CF and healthy gut mucosa

**Fig. 2:**
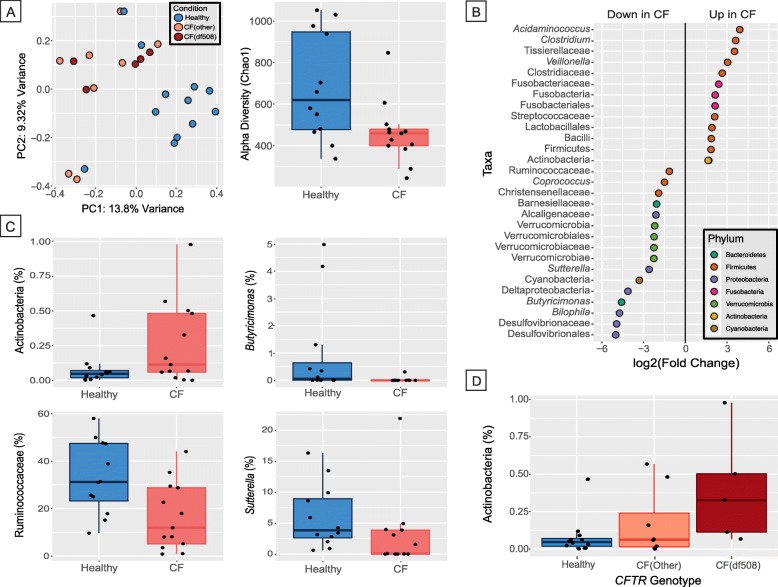
Differences between cystic fibrosis (CF) and healthy gut mucosal microbiota. **a** (left) Principal coordinate analysis plot based on Bray-Curtis distance indicating difference in beta-diversity between CF and healthy gut mucosal microbiome. The axes represent the percentage variance along the first two principal components and the color of samples indicates their mutation status, i.e., Healthy, CF (other), and CF (df508); (right) Boxplot depicting difference in alpha diversity (Chao1 metric) between CF and healthy gut microbiome. **b** Dotplot showing significantly differentially abundant OTUs (*q* value < 0.1), where OTUs are grouped by genera along the *y*-axis and colored by phylum. The *x*-axis indicates the log2 fold-change in CF compared to healthy as baseline. **c** Boxplots indicating the percentage relative abundance of taxa showing differential abundance between CF and healthy gut microbiome (*q* value < 0.1). **d** Boxplot depicting gradient-like trend in abundance for Actinobacteria for three genotypes—Healthy, CF (other), and CF (df508)

To further understand the potential of altered microbiota-host interaction in the CF colon, we next investigated differences in the composition of the mucosal microbiome between CF and healthy individuals. We used negative sequenced controls to verify that our downstream results were not affected by any potential contaminants (see “Methods”). We found a significant difference between beta-diversity of gut mucosal microbiome in CF patients compared to healthy individuals with respect to unweighted UniFrac and non-phylogenetic Bray-Curtis metrics (Adonis *p* value = 0.001). As observed in the PCoA plot (Fig. 2a), the samples were clustered based on their disease condition (CF or healthy). The overall biodiversity of mucosal microbiome was depleted in CF compared to healthy samples, which was depicted by a significant decrease in alpha diversity measured by Chao1 (*p* value = 0.015, Wilcoxon rank-sum test, Fig. 2a) and observed OTUs (*p* value = 0.024, Wilcoxon rank-sum test, in Additional file 2: Figure S15)) metrics in CF relative to healthy controls.

We assessed the changes in abundance of microbes at various taxonomic levels between CF and healthy gut mucosal microbiome using phyloseq. We found 51 OTUs that were significantly differentially abundant between CF and healthy individuals (*q* value < 0.1, Additional file 4). At different taxonomic ranks, we found 7 genera, 10 families, 4 orders, 4 classes, and 5 phyla differentially abundant between CF and healthy samples (*q* value < 0.1 by Wald’s test; Additional file 4). Overall, an increased abundance in taxa, predominantly belonging to Firmicutes (specifically *Clostridium*) and Fusobacteria, was observed in CF individuals compared to healthy controls, while taxa belonging to Bacteroidetes, Verrucomicrobia, and Proteobacteria phyla showed a marked decrease in patients with CF relative to healthy controls (Fig. 2b). In particular, there was an increase in abundance of class Actinobacteria in individuals with CF compared to healthy controls (*q* value = 0.079), while *Butyricimonas* (*q* value = 0.009), Ruminococcaceae (*q* value = 0.081), and *Sutterella* (*q* value = 0.040) were found depleted in CF samples (Fig. 2c). Additional examples of differentially abundant taxa between CF and healthy samples can be found in the Additional file 2: Figure S16).

Next, we tested whether *CFTR* genotype, which affects disease severity, is associated with variation in the microbiome. Specifically, we hypothesized that variation in the microbiome is correlated with the number of alleles of the DF508 mutation, a deletion of an entire codon within *CFTR* that is the most common cause for CF. To test this, we performed a likelihood ratio test to identify differentially abundant taxa between three genotype classes: CF-DF508 (homozygous for the DF508 mutation), CF-other (either one or zero copies of the DF508 mutation), and healthy (no known mutations in *CFTR*). We found a gradient-like trend in abundance for Actinobacteria (*q* value = 0.081), showing increase in abundance with increasing severity of mutation status (Fig. 2d).

To assess the potential functional changes in the microbiome, we predicted abundance of metabolic pathways and enzymes using the PICRUSt pipeline [31] and KEGG database and compared them for differences between CF and healthy individuals. Seven predicted pathways (as defined by KEGG level 3) were found to be differentially abundant between CF and healthy: bacterial toxins were enriched in CF compared to healthy, while propanoate metabolism, restriction enzyme, pantothenate and CoA biosynthesis, thiamine metabolism, amino acid-related enzymes, and aminoacyl-tRNA biosynthesis were depleted in CF compared to healthy (*q* value < 0.2 using Wilcoxon rank-sum test; in Additional file 2: Figure S17).

### Interactions between gastrointestinal cancer-related host genes and gut microbes

**Fig. 3:**
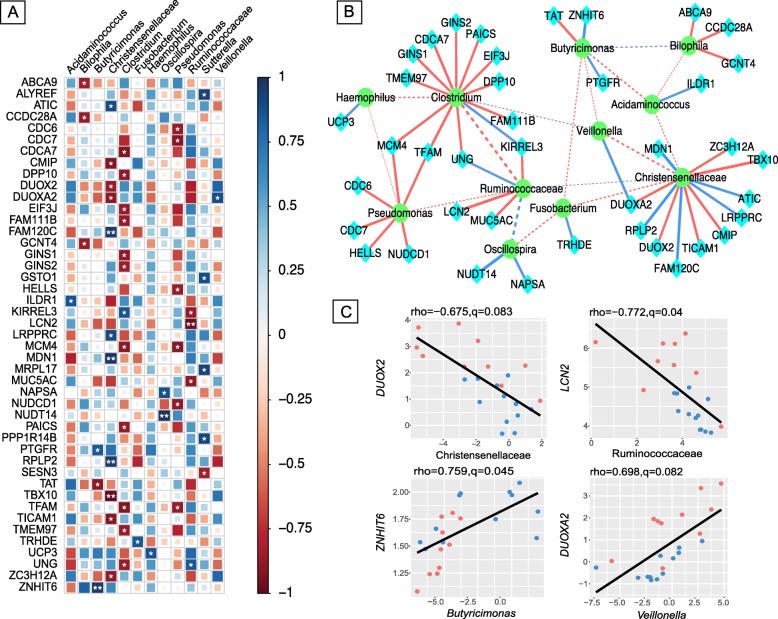
Interactions between genes associated with colorectal cancer and gut mucosal microbes. **a** Correlation plot depicting gene-microbe correlations. Color and size of the squares indicate the magnitude of the correlation, asterisks indicate significance of correlation (** indicates *q* value < 0.05 and * indicates *q* value < 0.1). **b** Network visualizing significant gene-microbe correlations (solid edges, *q* value < 0.1) and significant microbe-microbe correlations (dashed edges, SparCC |R| > =0.1 and *p* value < 0.05). Blue edges indicate positive correlation and red edges indicate negative correlation. Edge thickness represents the strength of the correlation. **c** Scatterplots depicting pattern of grouping by cystic fibrosis (red) and healthy (blue) samples in a few representative gene-microbe correlations, where the strength of correlation (Spearman rho) and significance (*q*) is indicated at the top of each plot

In order to investigate the relationship between host genes and microbes in the colonic mucosa and their potential role in the pathogenesis of gastrointestinal cancers in CF patients, we considered correlations between 250 differentially expressed genes enriched for GI cancers and 35 microbial taxa (collapsed at the genus or last characterized level and filtered at 0.1% relative abundance, see “Methods”). Using Spearman correlations, we found 50 significant unique gene-microbe correlations in the gut (*q* value < 0.1), where the magnitude of correlation (Spearman rho) ranged between − 0.77 and 0.79 (Additional file 5). Interestingly, most of the taxa that significantly correlated with the genes also differed significantly in abundance between CF and healthy individuals. We visualized all the correlations between taxa abundance and host gene expression in Fig. 3a. In particular, we found some significant positive gene-taxa correlations (*q* value < 0.05), between *Butyricimonas* and *ZNHIT6* (Spearman rho = 0.76), Christensenellaceae and *MDN1* (Spearman rho = 0.78), and *Oscillospira* and *NUDT14* (Spearman rho = 0.79). A few significant negative correlations (*q* value < 0.05), such as between Christensenellaceae and *TBX10* (Spearman rho = − 0.78), and Ruminococcaceae and *LCN2* (Spearman rho = − 0.77) were also found.

To characterize potential microbe-microbe interactions in our dataset, we computed correlations between the microbes significantly correlated (*q* value < 0.1) with the genes using SparCC (see “Methods” and Additional file 5) [35]. The notable aspects of the significant gene-microbe correlations (*q* value < 0.1) and significant microbe-microbe correlations (SparCC |R| > =0.1 and pseudo-*p* value < 0.05) are graphically represented in Fig. 3b, where solid edges denote gene-microbe correlations and dashed edges represent microbe-microbe correlations. This subnetwork of microbe-microbe correlations depicts correlated abundance changes in the microbiome as a function of their presence (Fig. 3b, dashed edges). For instance, *Bilophila* and *Butyricimonas* are both depleted in CF (*q* value < 0.05), and the abundance of the two genera is also correlated across individuals (SparCC R = 0.5, pseudo-*p* value = 0.04). On the other hand, Ruminococcaceae was found depleted in CF (*q* value = 0.081), while *Clostridium* was enriched in CF (*q* value = 0.0004), and this inverse co-occurrence pattern leads to a negative correlation between the two taxa across study participants (SparCC R = − 0.66, pseudo-*p* value = 0). Furthermore, in the gene-microbe subnetwork (Fig. 3b, solid edges), microbial nodes have more edges on average compared to genes, where Christensenellaceae and *Clostridium* formed distinct hubs in the network. This potentially implies that these microbes and their pathways are shared across multiple GI cancer-associated genes. Of note, *Bilophila*, *Clostridium*, and *Pseudomonas* are mostly negatively correlated with GI cancer genes, while *Haemophilus*, *Oscillospira*, *Veillonella*, *Fusobacterium*, and *Acidaminococcus* are only positively correlated with GI cancer genes (*q* value < 0.1).

In addition to the overall network, Fig. 3c depicts pairwise correlations between host gene expression and microbial taxa where both have been previously linked to CRC and thus may be of interest. For example, *LCN2*, known to be overexpressed in human CRC and other cancers [58], is negatively correlated with Ruminococcaceae (Spearman rho = − 0.77, *q* value = 0.040), which is found depleted in CRC [59, 60]. Both *DUOX2* and *DUOXA2* are found to be negatively correlated with Christensenellaceae (Spearman rho < − 0.65, *q* value < 0.1), while *DUOXA2* is positively correlated with *Veillonella* (Spearman rho = 0.70, *q* value = 0.082). *DUOX2* and its maturation factor *DUOXA2* are responsible for H_2_O_2_ production in human colon and are known to be upregulated in gastrointestinal inflammation [61, 62]. Christensenellaceae, a heritable taxon [63], has been shown to decrease in abundance in conventional adenoma [60], a precursor of CRC, whereas *Veillonella*, which is known to be proinflammatory, is found to be represented in human CRC [64]. Thus, the pattern of grouping by CF and healthy samples in these representative correlations are found to be similar to known associations in CRC and other gastrointestinal malignancies.

## Discussion

Recent advances in treatment have significantly prolonged the lives of CF patients [65]. However, this has led to new challenges, such as an elevated risk for gastrointestinal cancer [66]. Thus, CF patients show 5–10-fold increased risk of CRC compared to healthy individuals and that increases even further with immunosuppressive drugs [3, 6]. Understanding the molecular mechanisms that control the increased risk is key for early detection and the development of tailored treatments [6]. The importance of interactions between host and microbiome in the pathogenesis of colorectal cancer has become increasingly clear [59, 67]. To understand the role of these interactions in CF, we jointly profiled host colon gene expression and mucosal microbiome composition data in CF patients and healthy controls. We observed an enrichment of cancer-associated dysregulated genes—specifically colon cancer—in CF patients compared to healthy controls. We also observed a shift in the microbiome and identified taxa previously linked to colon cancer that varied in their abundance between CF and healthy individuals. We further found relevant correlations between these cancer-enriched genes and microbes that may illuminate the mechanisms of CRC development in CF patients.

Several previous studies have studied the role of host gene regulation in CF patients [5, 68]. While results from previous studies are based on either phenotypic observations, examining candidate genes such as *CFTR*, or an exploration of gene expression data from respiratory or blood samples [5, 69], our work is the first, as far as we know, that focused on a comprehensive transcriptomic analysis of colon biopsies. This allowed us to characterize patterns of host gene regulation specific to the CF colon epithelium. In addition to an enrichment of cancer-related pathways among genes that are differentially expressed in CF, we also observed an enrichment for immune response pathways, including signal transduction, cell adhesion, and viral infection. Interestingly, one of the most significant pathways enriched in our current data, the eIF2 signaling pathway, has been previously shown to play an important role in immune response, and cells with defective eIF2 signaling pathway were more susceptible to bacterial infections [70]. Furthermore, our analysis revealed that tumor suppressor genes are differentially regulated in the colon of CF patients. In addition to *CFTR*, we found other tumor suppressor genes, such as *HPGD*, to be downregulated in CF patients’ colon. *HPGD* was previously shown to be downregulated in the lungs of CF patients [5, 71]. Downregulation of these tumor suppressor genes can lead to predisposition of colon cancer [72]. Additionally, while we did see an enrichment of genes related to CRC pathway, we further tested these enrichments to see if this was a result of inflammation or high mucosal turnover in CF patients. No signatures of inflammation were found in our study when compared to the genes enriched in Crohn’s disease (CD) patients with inflammation [52]. This further suggests a potential mechanism underlying the reported increased risk and early development of colon cancer in CF patients [5, 66].

In addition to host gene regulation, the microbiome has also been implicated in the development of many diseases, including CRC [59, 73]. In the context of CF, previous studies have focused on characterizing shifts in the fecal or airway microbiome [14, 74]. Here, we profiled the colonic mucosal microbiome, with the goal of understanding its role in the development of CRC in CF patients. We found a clear distinction between microbiome populations from CF compared to healthy mucosa. Overall, similar to several other GI diseases, we also observed a reduced microbial biodiversity in the CF population [75]. We found an increase in Actinobacteria, one of the most predominant genera found in the sputum of CF patients [70], but decreased in colon cancer gut microbiome [73]. Furthermore, our observation of a significant decrease in the abundance of Verrucomicrobia, and increase in abundance of Firmicutes and Actinobacteria in CF patients, is consistent with the findings from the fecal microbiome of CF patients [17]. We also found a depletion in butyrate-producing bacteria, such as Ruminococcaceae and *Butyricimonas*, *s*imilar to previously reported depletion in butyrate-producing microbes by Manor et al. [14] in their study comparing CF fecal samples from children on varying degrees of fat intake. Butyrate helps promote growth and can also act as an anti-inflammatory agent and is therefore an important compound for colon health [14]. Interestingly, mice with compromised GI defense system also had a reduced number of butyrate-producing bacteria, similar to our observations in the CF patients, who generally consume a high-fat diet [76]. The loss in abundance of butyrate-producing Ruminococcaceae has also been previously observed in CRC [59, 77]. While the mechanism of *Clostridium* and *Fusobacterium* in tumorigenesis is yet to be defined, several studies have reported an increased presence of these two taxa in colon of CRC patients [78]. Interestingly, we also found an increase in these two previously known carcinogenic bacteria in CF patients. Thus, higher abundance of potentially pathogenic bacteria, such as *Clostridium* and Fusobacteria, combined with depletion of protective microbes, such as Ruminococcaceae, may facilitate carcinogenesis in the CF gut. Understanding the underlying mechanism of carcinogenesis can not only be useful for developing therapeutics, but potentially help define biomarkers for early detection of CRC in CF patients. Lastly, we found an increase in predicted bacterial toxins in the CF population, which might be explained by the increase in pathogenic bacteria such as *Pseudomonas* and *Veillonella.* This can potentially damage epithelial cells or induce mutations leading to unfavorable clinical outcome [79].

Integrating mucosal microbiome and host gene expression profiles, we observed several correlations between differentially expressed colon epithelial genes and gut mucosal bacteria in CF. Co-culture and obligate cross-feeding studies have shown an increased virulence of a pathogen in the presence of other bacteria, thus triggering an immune response that can determine the clinical outcome [80, 81]. One such example is the increased virulence of *Pseudomonas* in the presence of *Veillonella* as seen in a mice tumor model resulting in host clinical deterioration [81]. Interestingly, we found both of these microbes (*Veillonella* and *Pseudomonas*) in higher abundance in CF patients. However, we have also observed an example of the opposite pattern, showing a depletion in a downregulated pathogenic bacterium, *Bilophila*, in CF population compared to healthy controls. While *Bilophila* has previously been associated with CRC, its decrease in CF patients in our current study can be due to the lack of availability of necessary substrate, environmental conditions, or presence of other commensal rivals, which in our study might be *Acidaminococcus* due to its negative correlation with the pathogen [82]. Furthermore, we also found a strong correlation between *Veillonella* and *DUOXA2*, a highly expressed gene causing inflammation in ulcerative colitis [83]. Another such correlation that we observed was between highly expressed *LNC2* gene, which plays a role in innate immunity and has been previously found to be upregulated in human colon cancers [58], and depletion of Ruminococcaceae, a butyrate-producing bacteria that helps maintain colon health [14].

Our study has limitations. First, all CF patients have a substantial burden of antibiotic exposure. Since antibiotics affect the gut microbiome [84–86], this may impact the differences we observe between CF and healthy mucosal microbiome. Since the colonoscopies were done electively for colorectal cancer screening, none of the patients were being treated for acute infections. However, it is difficult to account for long-term effects of antibiotics as there is no comparable exposure in non-CF patients. Similarly, CF patients are also on a high-calorie diet that is high in protein and fat, this might be an additional factor impacting the microbiome. Furthermore, due to their inability to breakdown and absorb nutrients, the CF patients also have to supplement for pancreatic enzymes. Thus, our study considers the joint effects of diet, medication, and disease, as it is challenging to deconfound these effects in human studies of CF. Secondly, while some of the CF patients undergoing biopsy had polyps, none of them had developed tumors. It would be interesting to see if patients with tumors also show similar enrichments and correlation, which can help achieve a more comprehensive insight into the early development of CRC in CF patients. In addition, although we report a potential role for host gene-microbe and microbe-microbe interactions in the pathology of CRC, our study focused on correlations, and causality is not inferred. Considering that studying causality is challenging in humans, future studies using in vivo or in vitro models can be useful to study specific host gene-microbe connections, understand the mechanism, and disentangle the direction of interaction [87]**.**

## Conclusions

To summarize, we report an analysis of the mucosal microbiome and host gene expression in the gut of CF patients and healthy controls. We find downregulation of tumor suppressor genes, as well as upregulation of genes that play a role in immune response and cause inflammation. Furthermore, we observe a shift in microbiome with depletion in butyrate-producing bacteria that may help maintain colon health and increase in pathogenic strains in individuals with CF. Lastly, our study provides a set of candidate interactions between gut microbes and host genes in the CF gut. Our work sheds light on the role of host-microbiome interactions and their relevance for the early development of CRC in CF patients. Our results can provide clinicians and researchers with biomarkers that may potentially serve as targets for stratifying risk of CRC in patients with CF.

## Supplementary information

**Additional file 1:** Metadata for RNA-Seq and 16S rRNA data.

**Additional file 2:** **Figure S1.** Experimental pipeline. **Figure S2.** Quality control of RNA-seq data. **Figure S3.** Sample clustering. **Figure S4.** Primary genes defining the PC. **Figure S5.** Quality control of samples. **Figure S6.** Transcript abundance. **Figure S7.** Data fitting. **Figure S8.** Differentially expressed genes (DEGs). **Figure S9.** Data smoothing. **Figure S10.** PCoA plot for OTU table with negative controls and true samples. **Figure S11.** Histogram of prevalence-based scores assigned by decontam to each OTU. **Figure S12.** Validation of differentially expressed genes. **Figure S13.** Canonical pathways. **Figure S14.** Colorectal gene network. **Figure S15.** Alpha diversity for observed OTUs and Shannon metrics in CF samples compared to healthy samples. **Figure S16.** Randomly selected differentially abundant taxa between CF and Healthy conditions. **Figure S17.** Differentially abundant predicted metabolic pathways in CF samples compared to healthy.

**Additional file 3:** Differentially expressed genes.

**Additional file 4:** Differentially abundant taxa.

**Additional file 5:** Gene-microbe and microbe-microbe correlation.

## Abbreviations

CF: Cystic fibrosis
CRC: Colorectal cancer
GI: Gastrointestinal
FDR: False discovery rate
OTU: Operational taxonomic unit
PICRUSt: Phylogenetic Investigation of Communities by Reconstruction of Unobserved States
KEGG: Kyoto Encyclopedia of Genes and Genomes

## References

[1] O’Sullivan BP, Freedman SD (2009). Cystic fibrosis. Lancet.

[2] Maisonneuve P, Marshall BC, Knapp EA, Lowenfels AB (2013). Cancer risk in cystic fibrosis: a 20-year nationwide study from the United States. J Natl Cancer Inst.

[3] Yamada A, Komaki Y, Komaki F, Micic D, Zullow S, Sakuraba A (2018). Risk of gastrointestinal cancers in patients with cystic fibrosis: a systematic review and meta-analysis. Lancet Oncol.

[4] Niccum DE, Billings JL, Dunitz JM, Khoruts A (2016). Colonoscopic screening shows increased early incidence and progression of adenomas in cystic fibrosis. J Cyst Fibros.

[5] Than BLN, Linnekamp JF, Starr TK, Largaespada DA, Rod A, Zhang Y (2016). CFTR is a tumor suppressor gene in murine and human intestinal cancer. Oncogene.

[6] Hadjiliadis D, Khoruts A, Zauber AG, Hempstead SE, Maisonneuve P, Lowenfels AB (2018). Cystic Fibrosis Colorectal Cancer Screening Consensus Recommendations. Gastroenterology.

[7] Starr TK, Allaei R, Silverstein KAT, Staggs RA, Sarver AL, Bergemann TL (2009). A transposon-based genetic screen in mice identifies genes altered in colorectal cancer. Science.

[8] Norkina O, Kaur S, Ziemer D, De Lisle RC (2004). Inflammation of the cystic fibrosis mouse small intestine. Am J Physiol Gastrointest Liver Physiol.

[9] Jakab RL, Collaco AM, Ameen NA (2011). Physiological relevance of cell-specific distribution patterns of CFTR, NKCC1, NBCe1, and NHE3 along the crypt-villus axis in the intestine. Am J Physiol Gastrointest Liver Physiol.

[10] Barker N, Ridgway RA, van Es JH, van de Wetering M, Begthel H, van den Born M (2009). Crypt stem cells as the cells-of-origin of intestinal cancer. Nature.

[11] Lynch SV, Goldfarb KC, Wild YK, Kong W, De Lisle RC, Brodie EL (2013). Cystic fibrosis transmembrane conductance regulator knockout mice exhibit aberrant gastrointestinal microbiota. Gut Microbes.

[12] Duytschaever G, Huys G, Bekaert M, Boulanger L, De Boeck K, Vandamme P (2013). Dysbiosis of bifidobacteria and Clostridium cluster XIVa in the cystic fibrosis fecal microbiota. J Cyst Fibros.

[13] Schippa S, Iebba V, Santangelo F, Gagliardi A, De Biase RV, Stamato A (2013). Cystic fibrosis transmembrane conductance regulator (CFTR) allelic variants relate to shifts in faecal microbiota of cystic fibrosis patients. PLoS One.

[14] Manor O, Levy R, Pope CE, Hayden HS, Brittnacher MJ, Carr R (2016). Metagenomic evidence for taxonomic dysbiosis and functional imbalance in the gastrointestinal tracts of children with cystic fibrosis. Sci Rep.

[15] Burke DG, Fouhy F, Harrison MJ, Rea MC, Cotter PD, O’Sullivan O (2017). The altered gut microbiota in adults with cystic fibrosis. BMC Microbiol.

[16] Miragoli Francesco, Federici Sara, Ferrari Susanna, Minuti Andrea, Rebecchi Annalisa, Bruzzese Eugenia, Buccigrossi Vittoria, Guarino Alfredo, Callegari Maria Luisa (2016). Impact of cystic fibrosis disease on archaea and bacteria composition of gut microbiota. FEMS Microbiology Ecology.

[17] de Freitas MB, Moreira EAM, Tomio C, Moreno YMF, Daltoe FP, Barbosa E (2018). Altered intestinal microbiota composition, antibiotic therapy and intestinal inflammation in children and adolescents with cystic fibrosis. PLoS One.

[18] Andrews S (2010). FastQC: a quality control tool for high throughput sequence data.

[19] Bray NL, Pimentel H, Melsted P, Pachter L (2016). Near-optimal probabilistic RNA-seq quantification. Nat Biotechnol.

[20] Pimentel H, Bray NL, Puente S, Melsted P, Pachter L (2017). Differential analysis of RNA-seq incorporating quantification uncertainty. Nat Methods.

[21] Love MI, Huber W, Anders S (2014). Moderated estimation of fold change and dispersion for RNA-seq data with DESeq2. Genome Biol.

[22] Soneson C, Love MI, Robinson MD (2015). Differential analyses for RNA-seq: transcript-level estimates improve gene-level inferences. F1000Res.

[23] Durinck S, Spellman PT, Birney E, Huber W (2009). Mapping identifiers for the integration of genomic datasets with the R/Bioconductor package biomaRt. Nat Protoc..

[24] Huse SM, Dethlefsen L, Huber JA, Mark Welch D, Relman DA, Sogin ML (2008). Exploring microbial diversity and taxonomy using SSU rRNA hypervariable tag sequencing. PLoS Genet..

[25] Al-Ghalith GA, Hillmann B, Ang K, Shields-Cutler R, Knights D (2018). SHI7 is a Self-Learning pipeline for multipurpose Short-Read DNA quality control. mSystems.

[26] Al-Ghalith GA, Montassier E, Ward HN, Knights D (2016). NINJA-OPS: Fast Accurate Marker Gene Alignment Using Concatenated Ribosomes. PLoS Comput Biol..

[27] Davis NM, Proctor DM, Holmes SP, Relman DA, Callahan BJ (2018). Simple statistical identification and removal of contaminant sequences in marker-gene and metagenomics data. Microbiome..

[28] Oksanen J, Blanchet FG, Friendly M, Kindt R, Legendre P, McGlinn D et al. vegan: Community Ecology Package. R package version 2.4-5; 2017. https://CRAN.R-project.org/package=vegan.

[29] McMurdie PJ, Holmes S (2013). phyloseq: an R package for reproducible interactive analysis and graphics of microbiome census data. PLoS One.

[30] Johansen HK, Nir M, Koch C, Schwartz M, Høiby N (1991). Severity of cystic fibrosis in patients homozygous and heterozygous for ΔF508 mutation. Lancet..

[31] Langille MGI, Zaneveld J, Caporaso JG, McDonald D, Knights D, Reyes JA (2013). Predictive functional profiling of microbial communities using 16S rRNA marker gene sequences. Nat Biotechnol..

[32] Weiss Sophie, Van Treuren Will, Lozupone Catherine, Faust Karoline, Friedman Jonathan, Deng Ye, Xia Li Charlie, Xu Zhenjiang Zech, Ursell Luke, Alm Eric J, Birmingham Amanda, Cram Jacob A, Fuhrman Jed A, Raes Jeroen, Sun Fengzhu, Zhou Jizhong, Knight Rob (2016). Correlation detection strategies in microbial data sets vary widely in sensitivity and precision. The ISME Journal.

[33] Dabney A, Storey JD, Warnes GR (2010). qvalue: Q-value estimation for false discovery rate control. R package version [Internet].

[34] Wei T, Simko V (2013). corrplot: Visualization of a correlation matrix. R package version 0 73.

[35] Friedman J, Alm EJ (2012). Inferring correlation networks from genomic survey data. PLoS Comput Biol..

[36] Smoot ME, Ono K, Ruscheinski J, Wang P-L, Ideker T (2011). Cytoscape 2.8: new features for data integration and network visualization. Bioinformatics..

[37] Salter SJ, Cox MJ, Turek EM, Calus ST, Cookson WO, Moffatt MF (2014). Reagent and laboratory contamination can critically impact sequence-based microbiome analyses. BMC Biol..

[38] Davies JC (2002). Pseudomonas aeruginosa in cystic fibrosis: pathogenesis and persistence. Paediatr Respir Rev..

[39] Bhagirath AY, Li Y, Somayajula D, Dadashi M, Badr S, Duan K (2016). Cystic fibrosis lung environment and Pseudomonas aeruginosa infection. BMC Pulm Med..

[40] Agnarsson U, Glass S, Govan JR (1989). Fecal isolation of Pseudomonas aeruginosa from patients with cystic fibrosis. J Clin Microbiol..

[41] Flo TH, Smith KD, Sato S, Rodriguez DJ, Holmes MA, Strong RK (2004). Lipocalin 2 mediates an innate immune response to bacterial infection by sequestrating iron. Nature..

[42] Wu X-L, Yang Z-W, He L, Dong P-D, Hou M-X, Meng X-K (2017). RRS1 silencing suppresses colorectal cancer cell proliferation and tumorigenesis by inhibiting G2/M progression and angiogenesis. Oncotarget..

[43] Berens EB, Sharif GM, Schmidt MO, Yan G, Shuptrine CW, Weiner LM (2017). Keratin-associated protein 5-5 controls cytoskeletal function and cancer cell vascular invasion. Oncogene..

[44] Bu P, Chen K-Y, Xiang K, Johnson C, Crown SB, Rakhilin N (2018). Aldolase B-mediated fructose metabolism drives metabolic reprogramming of colon cancer Liver Metastasis. Cell Metab.

[45] Kumara HMCS, Bellini GA, Caballero OL, Herath SAC, Su T, Ahmed A (2017). P-Cadherin (CDH3) is overexpressed in colorectal tumors and has potential as a serum marker for colorectal cancer monitoring. Oncoscience..

[46] Zhu H, Dougherty U, Robinson V, Mustafi R, Pekow J, Kupfer S, et al. EGFR signals downregulate tumor suppressors miR-143 and miR-145 in Western diet–promoted murine colon cancer: role of G1 regulators. Mol Cancer Res [Internet]. American Association for Cancer Research; 2011 [cited 2019 Jan 10]; Available from: http://mcr.aacrjournals.org/content/early/2011/07/01/1541-7786.MCR-10-0531.short. 10.1158/1541-7786.MCR-10-0531 PMC3819602 21653642

[47] Romero M, Sabaté-Pérez A, Francis VA, Castrillón-Rodriguez I, Díaz-Ramos Á, Sánchez-Feutrie M (2018). TP53INP2 regulates adiposity by activating β-catenin through autophagy-dependent sequestration of GSK3β. Nat Cell Biol..

[48] Yao L, Tak YG, Berman BP, Farnham PJ (2014). Functional annotation of colon cancer risk SNPs. Nat Commun..

[49] Dong Q, Meng P, Wang T, Qin W, Qin W, Wang F (2010). MicroRNA let-7a inhibits proliferation of human prostate cancer cells in vitro and in vivo by targeting E2F2 and CCND2. PLoS One..

[50] Mazzoccoli G, Pazienza V, Panza A, Valvano MR, Benegiamo G, Vinciguerra M (2012). ARNTL2 and SERPINE1: potential biomarkers for tumor aggressiveness in colorectal cancer. J Cancer Res Clin Oncol..

[51] Yu K, Lujan R, Marmorstein A, Gabriel S, Hartzell HC (2010). Bestrophin-2 mediates bicarbonate transport by goblet cells in mouse colon. J Clin Invest..

[52] Hong Sung Noh, Joung Je-Gun, Bae Joon Seol, Lee Chan Soo, Koo Ja Seol, Park Soo Jung, Im Jong Pil, Kim You Sun, Kim Ji Won, Park Woong Yang, Kim Young-Ho (2017). RNA-seq Reveals Transcriptomic Differences in Inflamed and Noninflamed Intestinal Mucosa of Crohnʼs Disease Patients Compared with Normal Mucosa of Healthy Controls. Inflammatory Bowel Diseases.

[53] Ham J, Costa C, Sano R, Lochmann TL, Sennott EM, Patel NU (2016). Exploitation of the apoptosis-primed state of MYCN-amplified neuroblastoma to develop a potent and specific targeted therapy combination. Cancer Cell..

[54] Vogelstein B, Fearon ER, Hamilton SR, Kern SE, Preisinger AC, Leppert M (1988). Genetic alterations during colorectal-tumor development. N Engl J Med..

[55] Chellappa K, Robertson GR, Sladek FM (2012). HNF4α: a new biomarker in colon cancer?. Biomark Med..

[56] Hünten S, Hermeking H (2015). p53 directly activates cystatin D/CST5 to mediate mesenchymal-epithelial transition: a possible link to tumor suppression by vitamin D3. Oncotarget..

[57] Feber A, Clark J, Goodwin G, Dodson AR, Smith PH, Fletcher A (2004). Amplification and overexpression of E2F3 in human bladder cancer. Oncogene..

[58] Maier HT, Aigner F, Trenkwalder B, Zitt M, Vallant N, Perathoner A (2014). Up-regulation of neutrophil gelatinase-associated lipocalin in colorectal cancer predicts poor patient survival. World J Surg..

[59] Burns MB, Lynch J, Starr TK, Knights D, Blekhman R (2015). Virulence genes are a signature of the microbiome in the colorectal tumor microenvironment. Genome Med..

[60] Peters BA, Dominianni C, Shapiro JA, Church TR, Wu J, Miller G (2016). The gut microbiota in conventional and serrated precursors of colorectal cancer. Microbiome..

[61] Wu Y, Antony S, Juhasz A, Lu J, Ge Y, Jiang G (2011). Up-regulation and sustained activation of Stat1 are essential for interferon-gamma (IFN-gamma)-induced dual oxidase 2 (Duox2) and dual oxidase A2 (DuoxA2) expression in human pancreatic cancer cell lines. J Biol Chem..

[62] Wu Y, Antony S, Hewitt SM, Jiang G, Yang SX, Meitzler JL (2013). Functional activity and tumor-specific expression of dual oxidase 2 in pancreatic cancer cells and human malignancies characterized with a novel monoclonal antibody. Int J Oncol..

[63] Goodrich JK, Waters JL, Poole AC, Sutter JL, Koren O, Blekhman R (2014). Human genetics shape the gut microbiome. Cell..

[64] Geng J, Song Q, Tang X, Liang X, Fan H, Peng H (2014). Co-occurrence of driver and passenger bacteria in human colorectal cancer. Gut Pathog..

[65] Cohen-Cymberknoh M, Shoseyov D, Kerem E (2011). Managing cystic fibrosis: strategies that increase life expectancy and improve quality of life. Am J Respir Crit Care Med..

[66] Hegagi M, Aaron SD, James P, Goel R, Chatterjee A (2017). Increased prevalence of colonic adenomas in patients with cystic fibrosis. J Cyst Fibros..

[67] Hurwitz BL (2018). 28 The relationship of host genetics and the microbiome in colon cancer. J Anim Sci..

[68] Kormann MSD, Dewerth A, Eichner F, Baskaran P, Hector A, Regamey N (2017). Transcriptomic profile of cystic fibrosis patients identifies type I interferon response and ribosomal stalk proteins as potential modifiers of disease severity. PLoS One..

[69] Tata M, Wolfinger MT, Amman F, Roschanski N, Dötsch A, Sonnleitner E (2016). RNASeq based transcriptional profiling of pseudomonas aeruginosa PA14 after short- and long-term anoxic cultivation in synthetic cystic fibrosis sputum medium. PLoS One..

[70] Shrestha N, Bahnan W, Wiley DJ, Barber G, Fields KA, Schesser K (2012). Eukaryotic initiation factor 2 (eIF2) signaling regulates proinflammatory cytokine expression and bacterial invasion. J Biol Chem..

[71] Wu Y‘a, Wang X, Wu F, Huang R, Xue F, Liang G (2012). Transcriptome profiling of the cancer, adjacent non-tumor and distant normal tissues from a colorectal cancer patient by deep sequencing. PLoS One..

[72] Myung S-J, Rerko RM, Yan M, Platzer P, Guda K, Dotson A (2006). 15-Hydroxyprostaglandin dehydrogenase is an in vivo suppressor of colon tumorigenesis. Proc Natl Acad Sci U S A..

[73] O’Keefe SJ (2014). Abstract SS01-01: The microbiome and colon cancer risk. Cancer Epidemiol Biomarkers Prev Am Assoc Cancer Res.

[74] Moran Losada Patricia, Chouvarine Philippe, Dorda Marie, Hedtfeld Silke, Mielke Samira, Schulz Angela, Wiehlmann Lutz, Tümmler Burkhard (2016). The cystic fibrosis lower airways microbial metagenome. ERJ Open Research.

[75] Tilg H, Kaser A (2011). Gut microbiome, obesity, and metabolic dysfunction. J Clin Invest..

[76] Hildebrandt MA, Hoffmann C, Hamady M, Chen Y-Y, Knight R, Bushman FD (2009). 662 High fat diet determines the composition of the gut microbiome independent of host genotype and phenotype. Gastroenterology.

[77] Mangifesta M, Mancabelli L, Milani C, Gaiani F, de’ Angelis N, de’ Angelis GL (2018). Mucosal microbiota of intestinal polyps reveals putative biomarkers of colorectal cancer. Sci Rep.

[78] Fukugaiti MH, Ignacio A, Fernandes MR, Ribeiro Júnior U, Nakano V, Avila-Campos MJ (2015). High occurrence of Fusobacterium nucleatum and Clostridium difficile in the intestinal microbiota of colorectal carcinoma patients. Braz J Microbiol..

[79] Barbieri JT. Bacterial toxins that modify the epithelial cell barrier. Bacterial-Epithelial Cell Cross-Talk: Molecular Mechanisms in Pathogenesis. Cambridge: Cambridge University Press; 2006. p. 184–210.

[80] Adamowicz EM, Flynn J, Hunter RC, Harcombe WR (2018). Cross-feeding modulates antibiotic tolerance in bacterial communities. ISME J..

[81] Pustelny C, Komor U, Pawar V, Lorenz A, Bielecka A, Moter A (2015). Contribution of Veillonella parvula to Pseudomonas aeruginosa-mediated pathogenicity in a murine tumor model system. Infect Immun..

[82] Kitamoto S, Nagao-Kitamoto H, Kuffa P, Kamada N (2016). Regulation of virulence: the rise and fall of gastrointestinal pathogens. J Gastroenterol..

[83] MacFie TS, Poulsom R, Parker A. DUOX2 and DUOXA2 form the predominant enzyme system capable of producing the reactive oxygen species H2O2 in active ulcerative colitis and are …. Inflamm Bowel Dis [Internet]. academic.oup.com; 2014; Available from: https://academic.oup.com/ibdjournal/article-abstract/20/3/514/4579005. 10.1097/01.MIB.0000442012.45038.0e 24492313

[84] Francino MP (2015). Antibiotics and the human gut microbiome: dysbioses and accumulation of resistances. Front Microbiol..

[85] Jakobsson HE, Jernberg C, Andersson AF, Sjölund-Karlsson M, Jansson JK, Engstrand L (2010). Short-term antibiotic treatment has differing long-term impacts on the human throat and gut microbiome. PLoS One..

[86] Lewis JD, Chen EZ, Baldassano RN, Otley AR, Griffiths AM, Lee D (2015). Inflammation, antibiotics, and diet as environmental stressors of the gut microbiome in pediatric Crohn’s disease. Cell Host Microbe..

[87] Luca F, Kupfer SS, Knights D, Khoruts A, Blekhman R (2018). Functional genomics of host–microbiome interactions in humans. Trends Genet..

