# Supplementary material for: Interactions between the gut microbiome and host gene regulation in cystic fibrosis
Source: Genome Med. 2020 Jan 28;12:12. doi: 10.1186/s13073-020-0710-2 (PMC6988342; doi:10.1186/s13073-020-0710-2)
Supplement: Supplementary file 2 — Additional file 2: Figure S1. Experimental pipeline. Figure S2. Quality control of RNA-seq data. Figure S3. Sample clustering. Figure S4. Primary genes defining the PC. Figure S5. Quality control of samples. Figure S6. Transcript abundance. Figure S7. Data fitting. Figure S8. Differentially expressed genes (DEGs). Figure S9. Data smoothing. Figure S10. PCoA plot for OTU table with negative controls and true samples. Figure S11. Histogram of prevalence-based scores assigned by decontam to each OTU. Figure S12. Validation of differentially expressed genes. Figure S13. Canonical pathways. Figure S14. Colorectal gene network. Figure S15. Alpha diversity for observed OTUs and Shannon metrics in CF samples compared to healthy samples. Figure S16. Randomly selected differentially abundant taxa between CF and Healthy conditions. Figure S17. Differentially abundant predicted metabolic pathways in CF samples compared to healthy. [file 13073_2020_710_MOESM2_ESM.pdf]

Interactions between the gut microbiome and host gene regulation in cystic fibrosis

Gargi Dayama[1,*], Sambhawa Priya[1,*], David E. Niccum[2], Alexander Khoruts[2,3,&], Ran Blekhman[1,4,&]

[1] Department of Genetics, Cell Biology and Development, University of Minnesota, Minneapolis, MN

[2] Department of Medicine, University of Minnesota, Minneapolis, MN

[3] Center for Immunology; BioTechnology Institute, University of Minnesota, Minneapolis, MN

[4] Department of Ecology, Evolution, and Behavior, University of Minnesota, Minneapolis, MN

* these authors contributed equally

[&] to whom correspondence should be addressed: khoru001@umn.edu (AK), blekhman@umn.edu (RB)

Supplementary Figures:

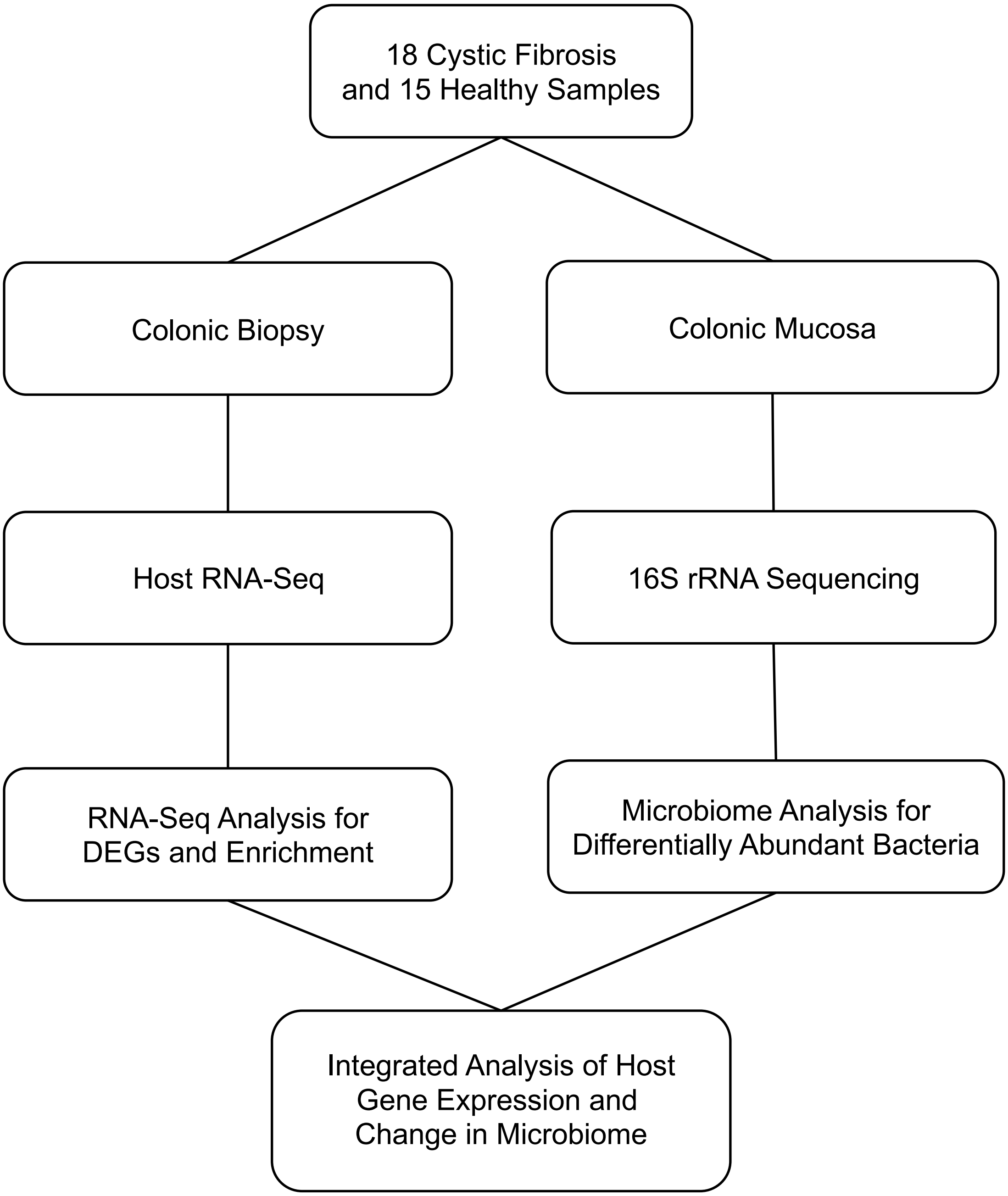

Fig S1. Experimental pipeline. Flowchart to illustrate major steps of the study, including sample information, host RNA-Seq analysis, microbiome analysis and integrated analysis.

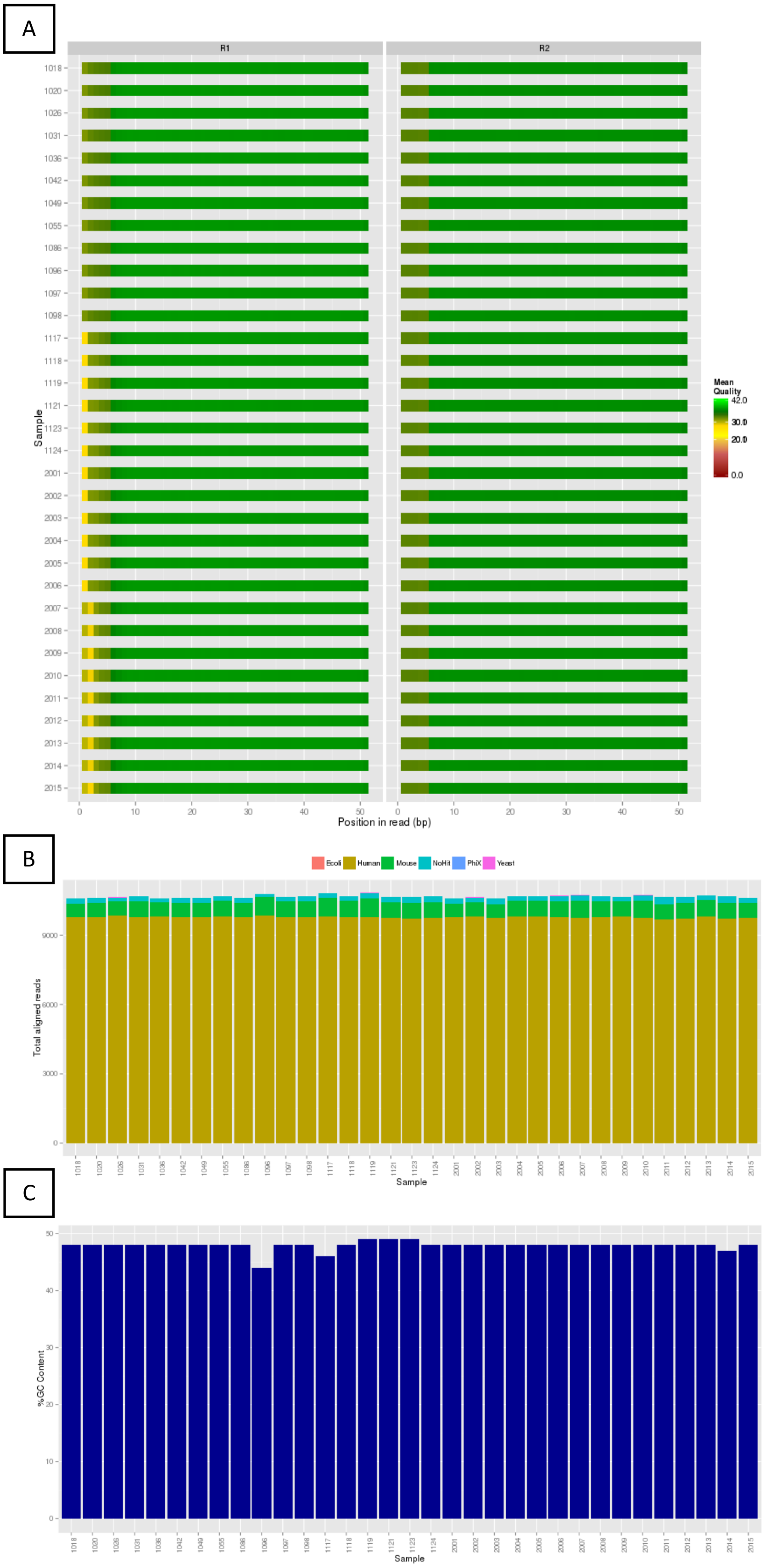

Fig S2. Quality control of RNA-seq data. (A) Average phred score of the forward (R1) and reverse (R2) reads, (B) Number of reads mapped to human and other species, (C) Total number of reads per sample

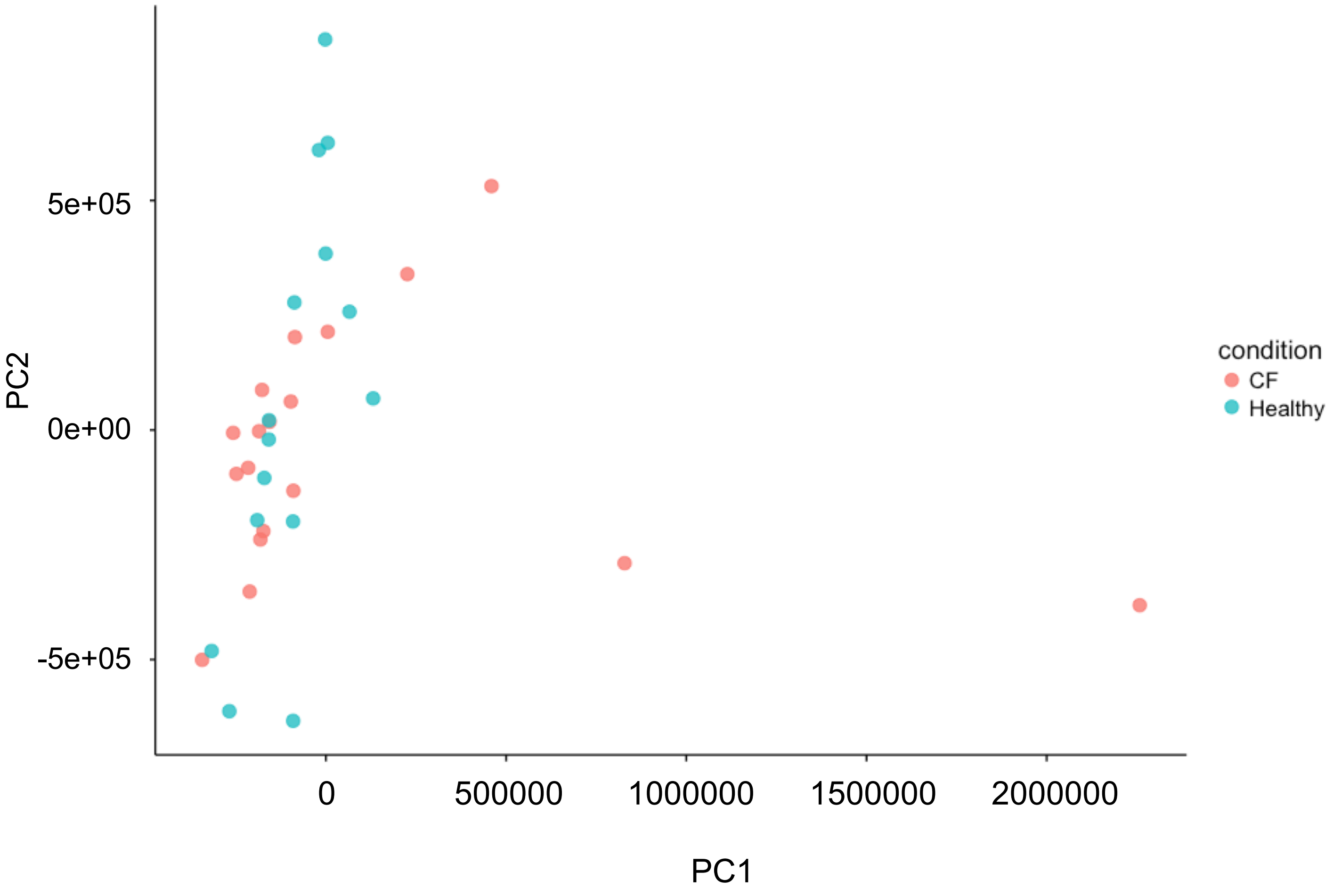

Fig S3. Sample clustering. PCA plot colored by condition, cystic fibrosis samples in red and healthy samples in green

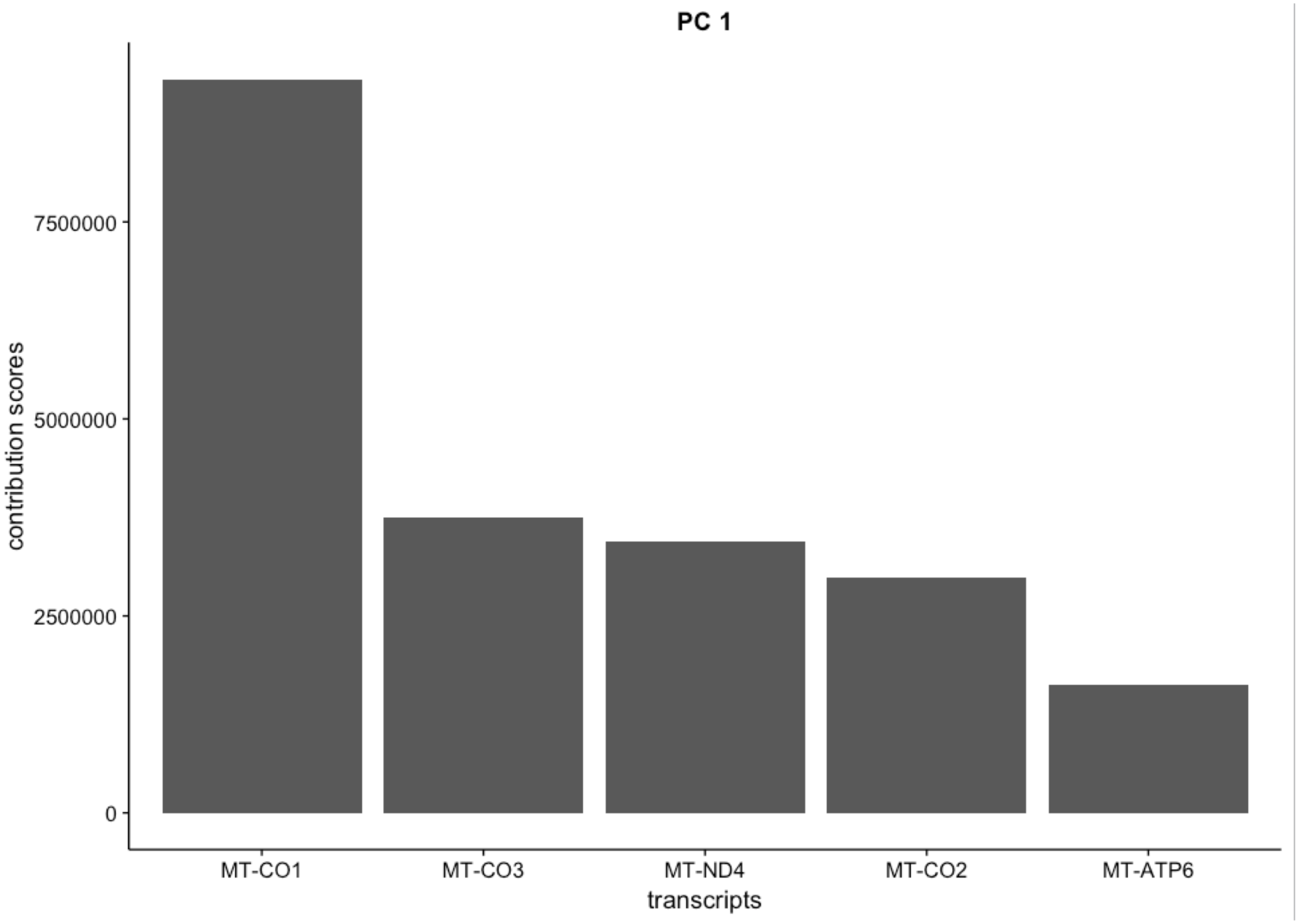

Fig S4. Primary genes defining the PC. Bar plots representing the top 5 genes driving PC1

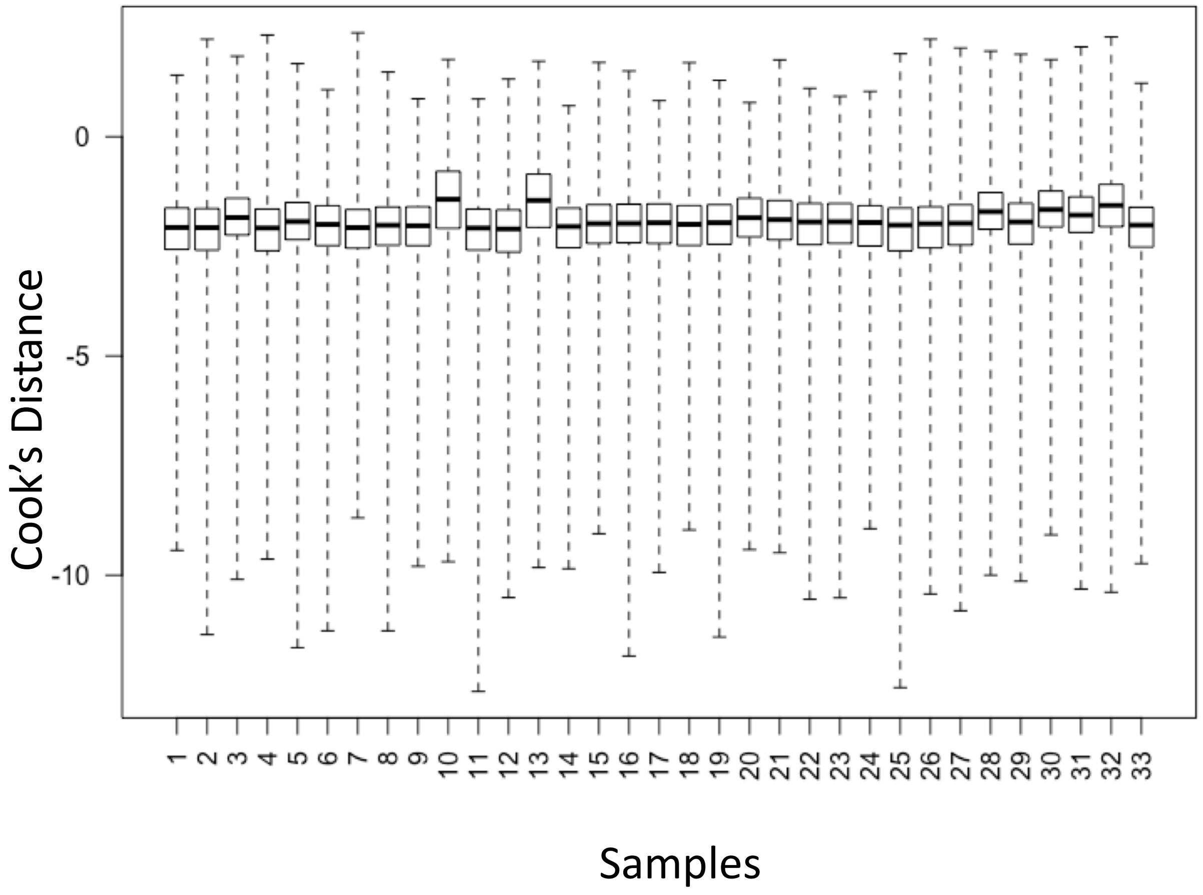

Fig S5. Quality control of samples. Box plots generated using cook's distance for determining any outlier samples

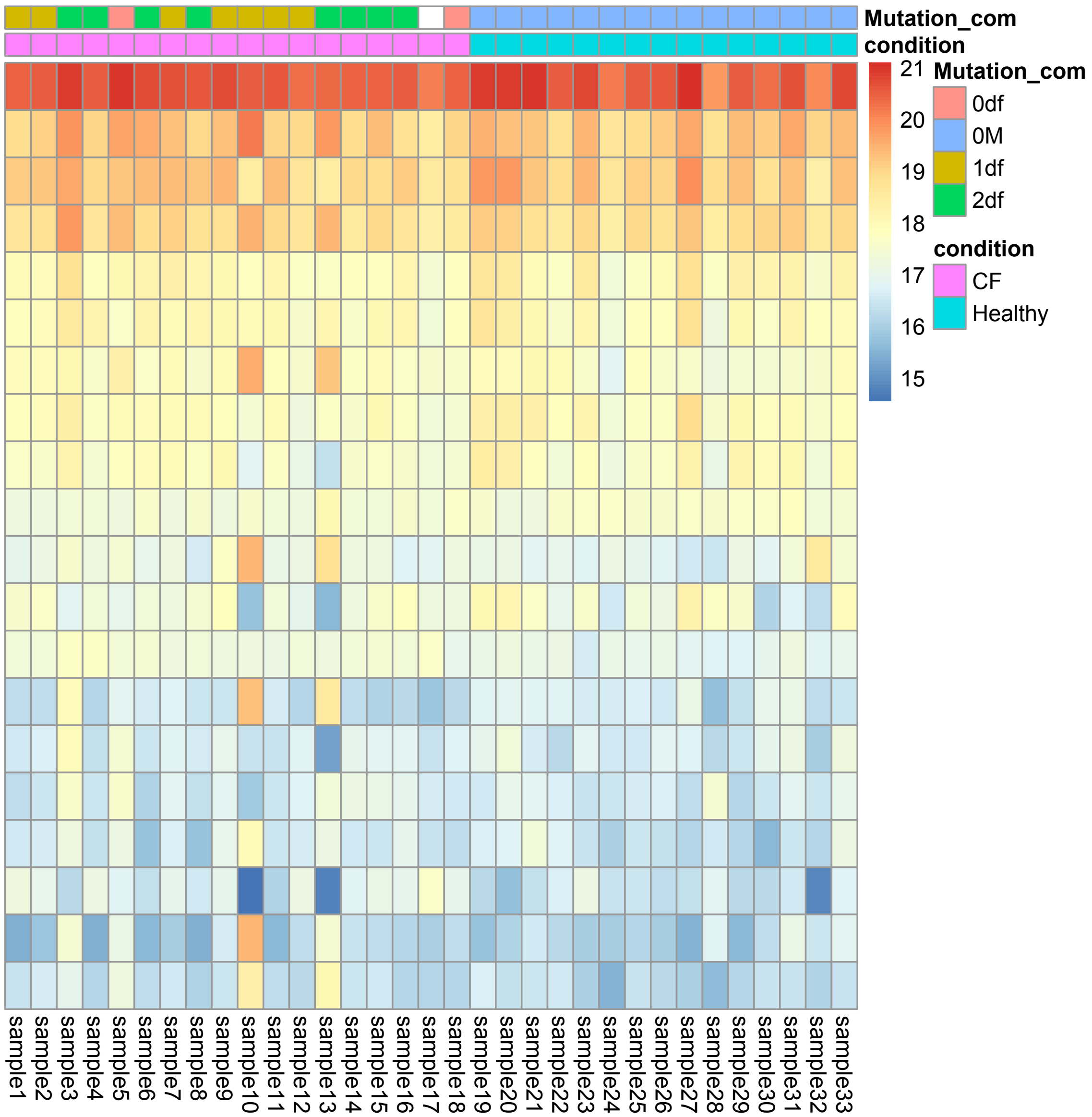

Fig S6. Transcript abundance. Heat map of top 20 genes, clustered by condition (cystic fibrosis represented by pink and healthy by cyan). Also includes the df508 mutation information on the CFTR gene (0M is for no mutation, 0df is when there is a mutation other than df508 deletion, 1df is for heterozygous df508 deletion and 2df is when both copies are deleted). Sample 17 did not have genotypic information.

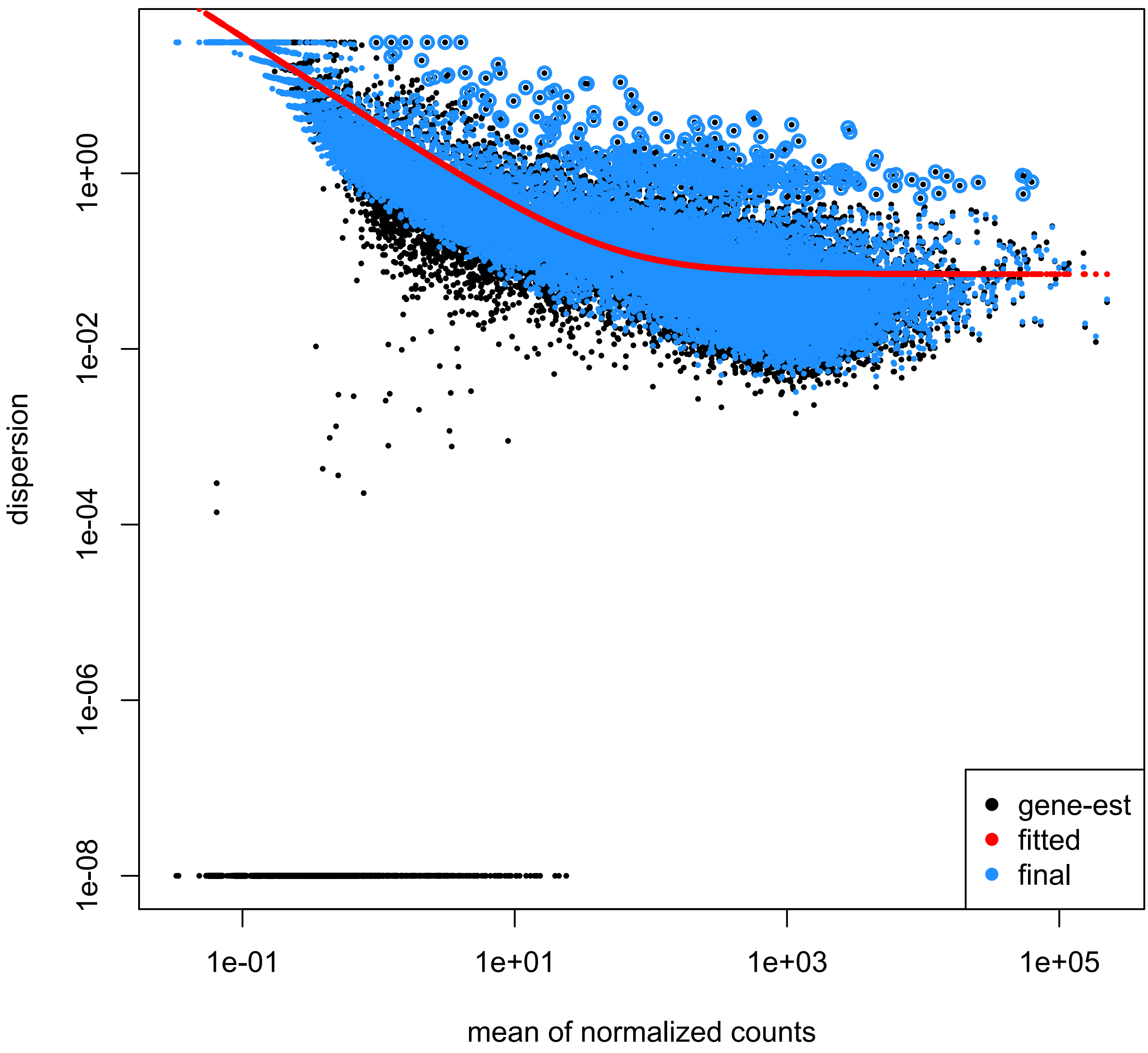

Fig S7. Data fitting. Dispersion plot for gene-wise estimates shrunken towards the fitted estimates (red line) and the outliers that were not shrunk are represented by the blue circles around the data points.

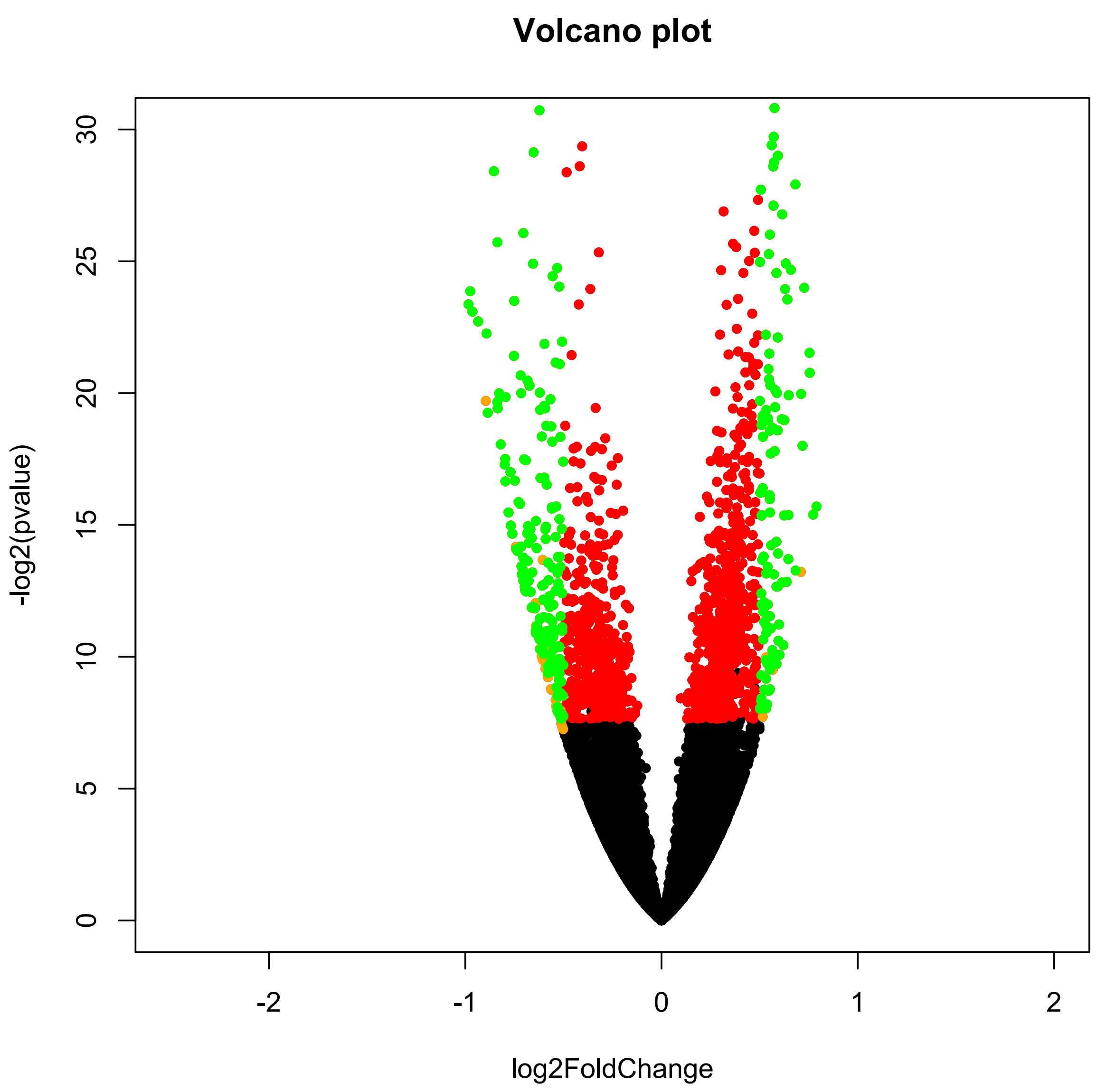

Fig S8. Differentially expressed genes (DEGs). Volcano plot showing all the DEGs, FDR < 0.05 represented by the red points and log fold change (log2|FC|) > 0.5 represented by green points.

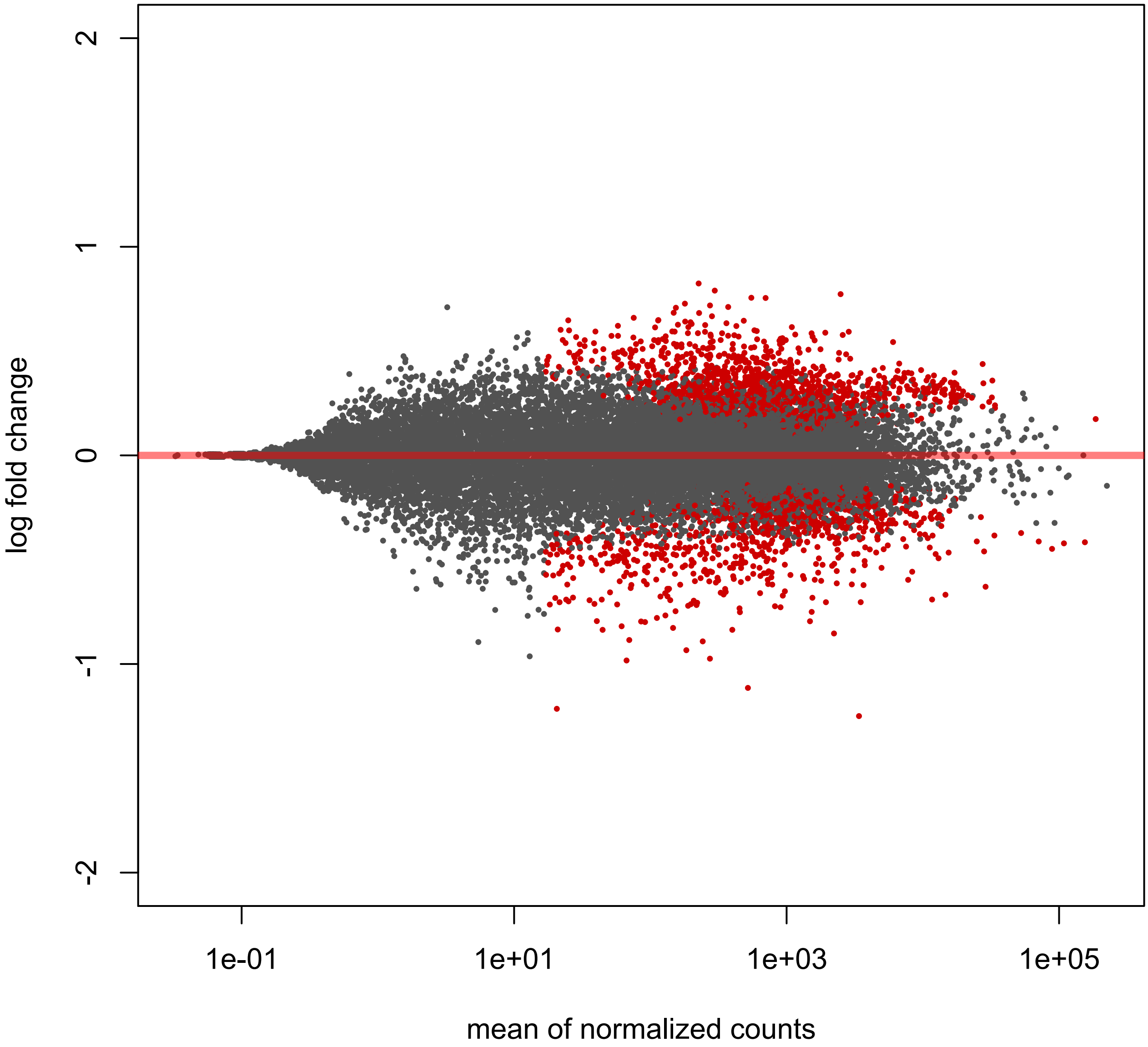

Fig S9. Data smoothing. MA plot showing the mean of normalized counts for all the transcripts. The red points are transcripts with large fold change due to low counts.

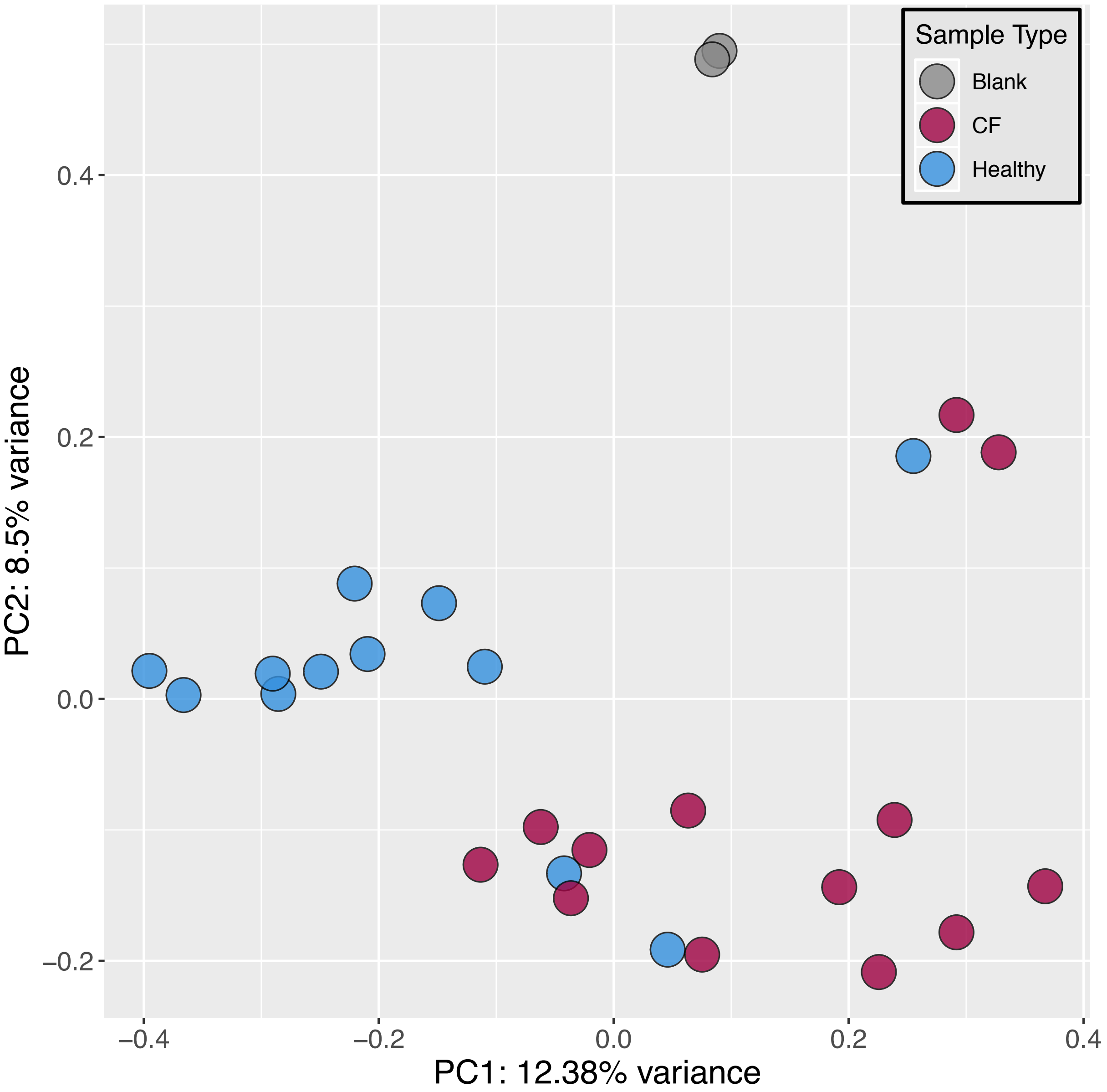

Fig S10. PCoA plot for OTU table with negative controls and true samples. The x-axis represents principal component 1, and y-axis shows principal component 2. The negative control (“blank”) samples are shown in grey, CF samples are in red and healthy samples in blue.

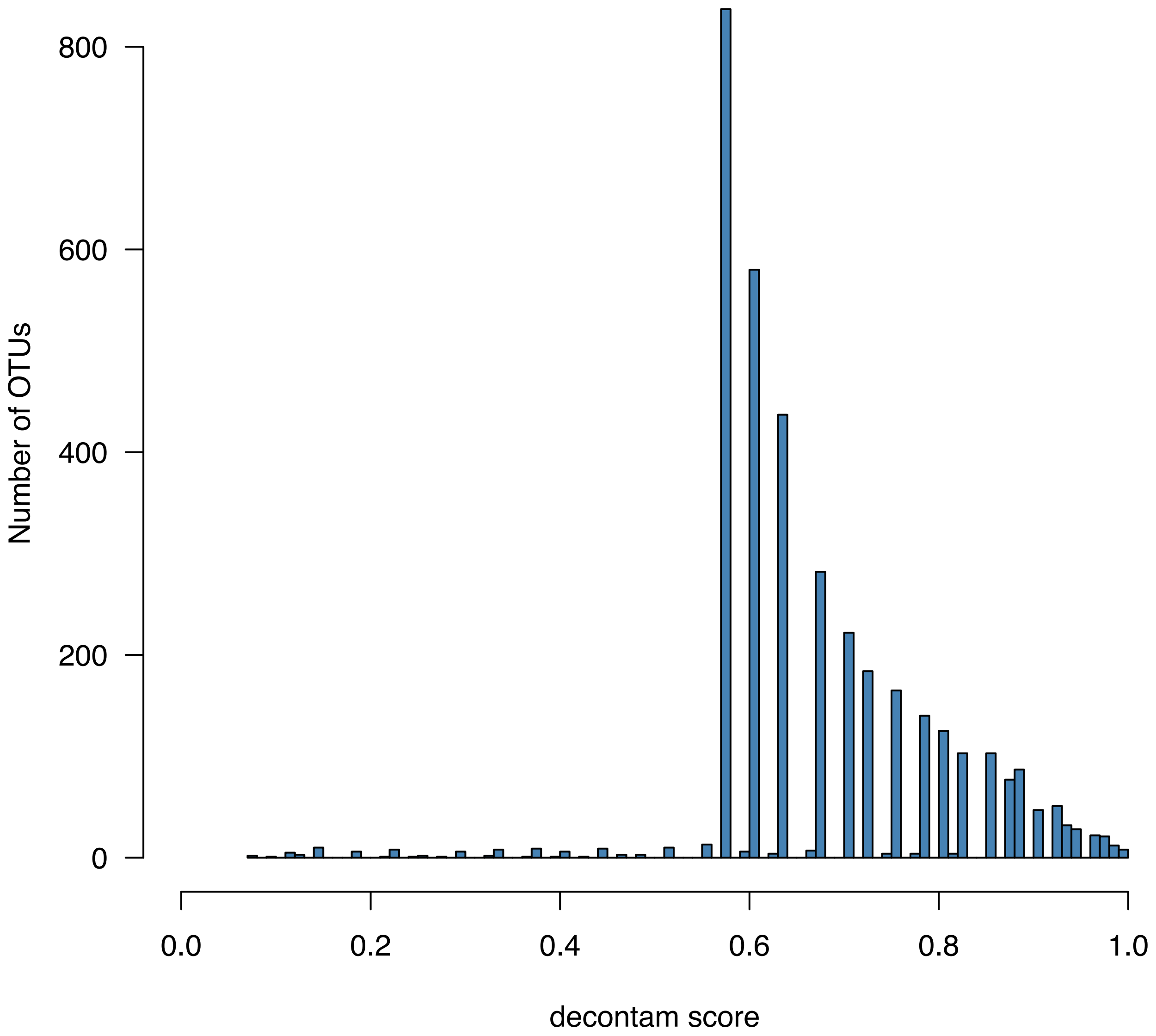

Fig S11. Histogram of prevalence-based scores assigned by decontam to each OTU. The x-axis represents the prevalence-based score assigned by decontam, and y-axis shows the number of OTUs assigned a given score.

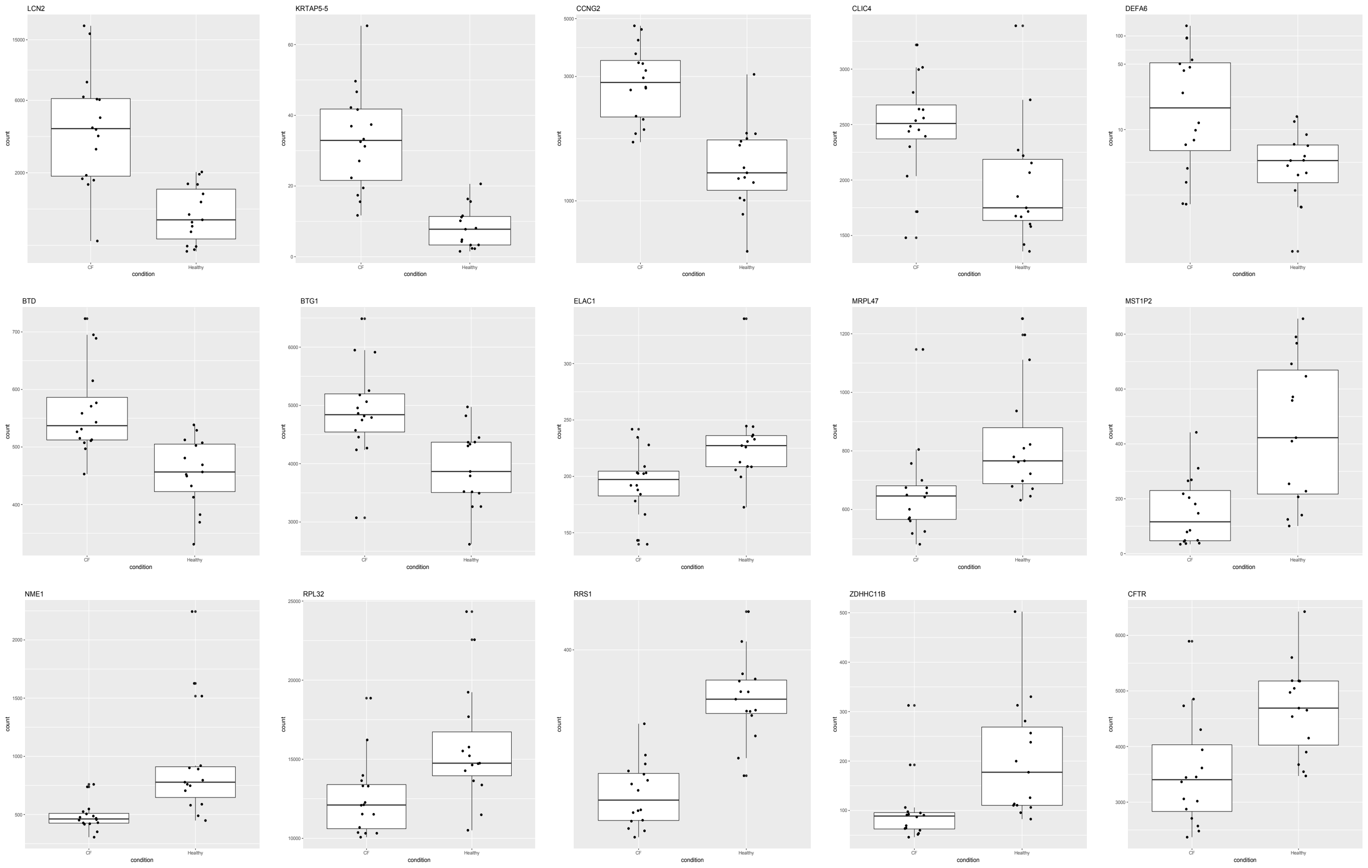

Fig S12. Validation of differentially expressed genes. Box plots of randomly selected DEGs, to show variability of counts between CF and healthy samples.

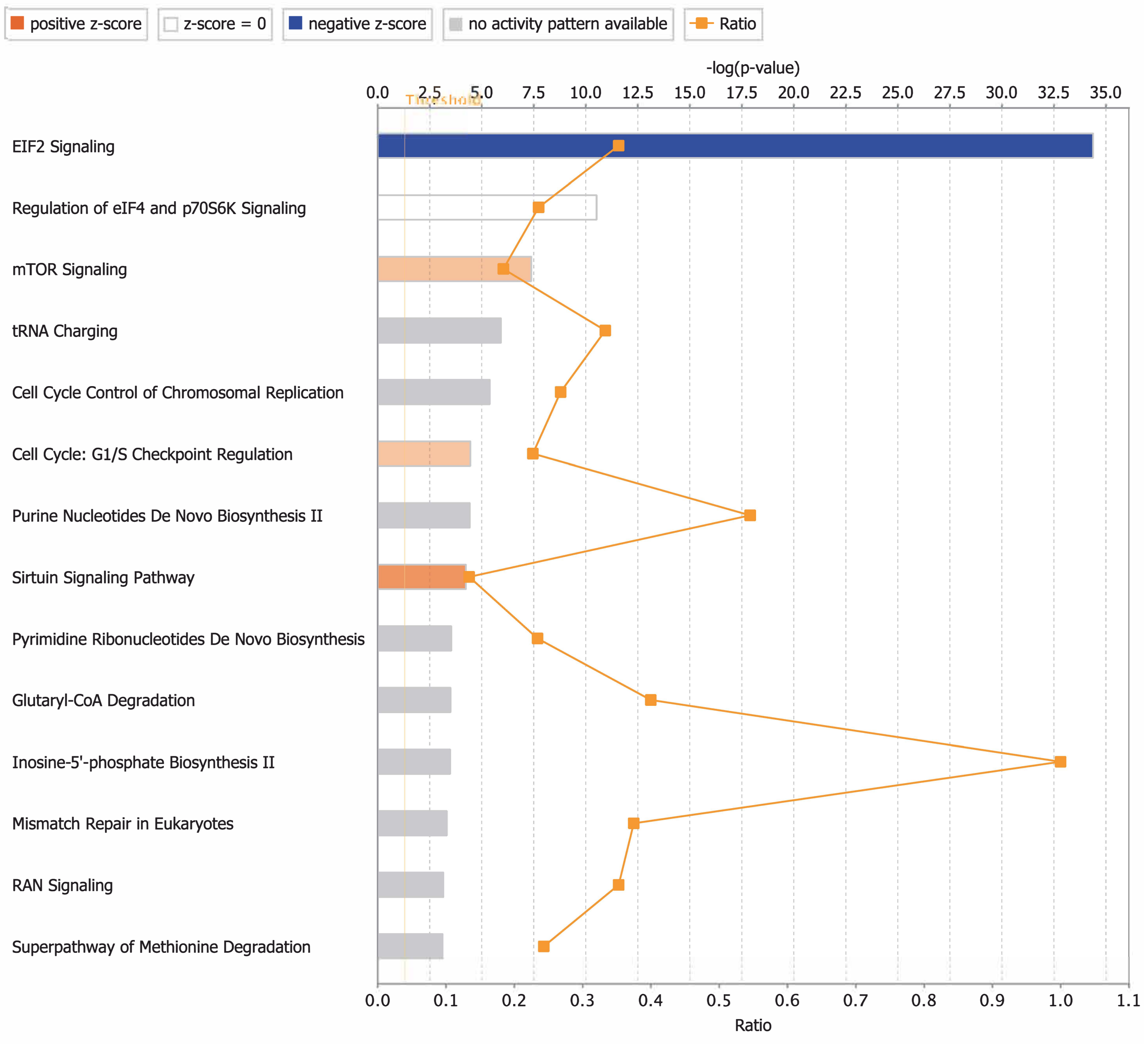

Fig S13. Canonical pathways. Bar plots showing select top pathways based on the negative log p-values for which the DEGs are enriched for. The blue bars are for pathways with negative z-score, while orange bars are for positive z-scores. Furthermore, if the z-score is either zero or has no activity pattern available then those bars are represented by white and grey bars respectively. The orange squares connected by the lines are to show the ratio.

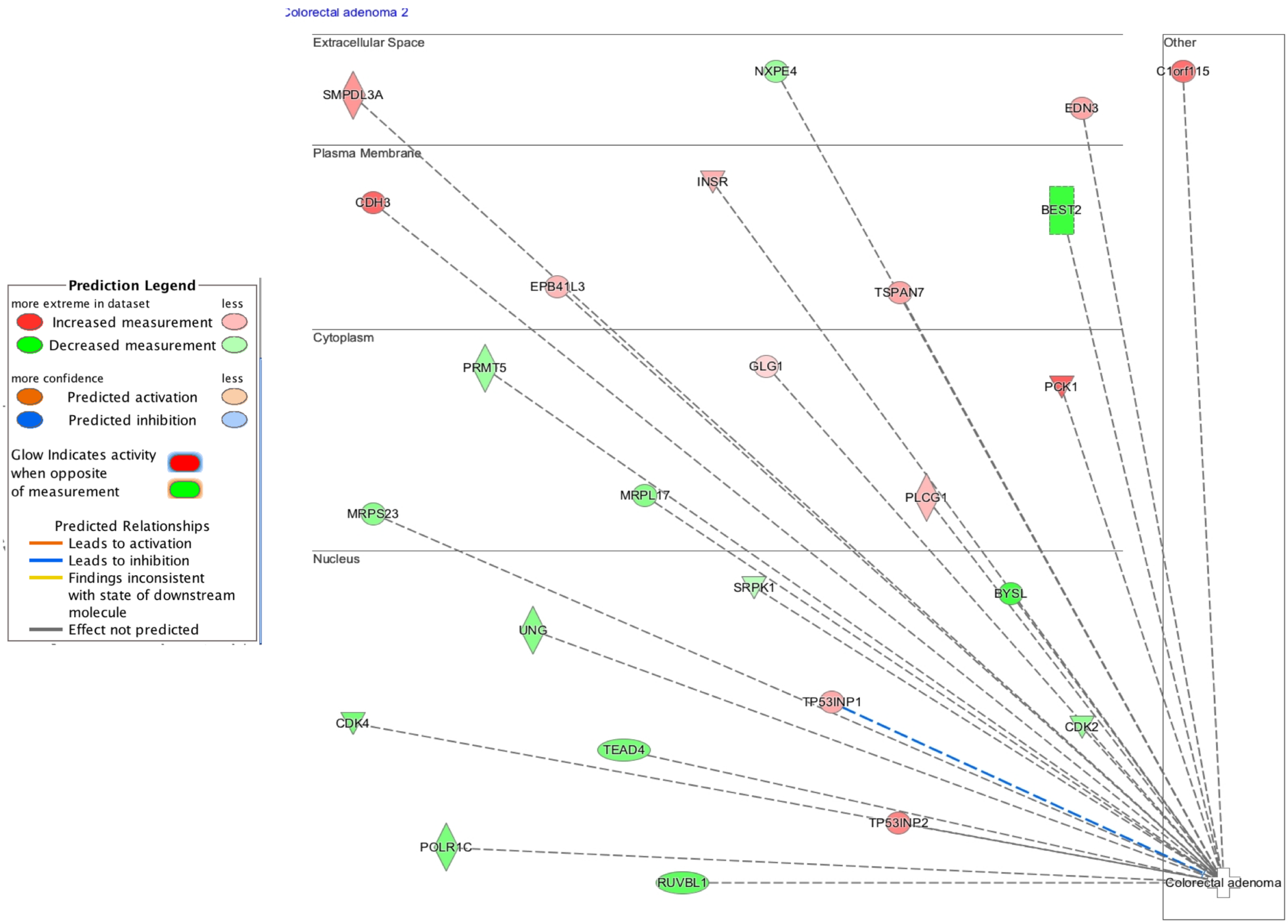

Fig S14. Colorectal gene network. A network of various genes involved in the colorectal cancer disease pathway. The genes have been separated into various bins based on the part of the cell in which they are most active. The upregulated genes in CF are represented by red color, while the downregulated genes are green.

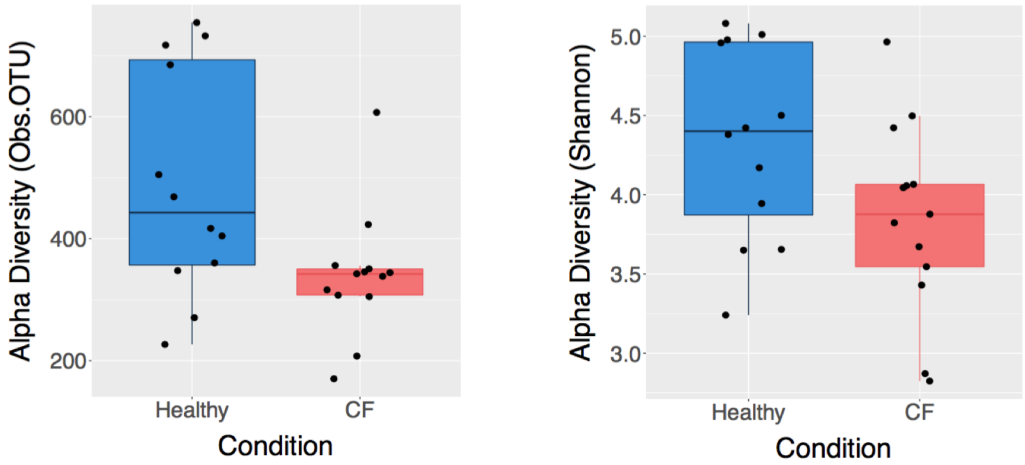

Fig S15. Alpha diversity for observed OTUs and Shannon metrics, is reduced in CF samples compared to healthy samples (p-value 0.024 and 0.087, respectively, Wilcoxon rank sum test)

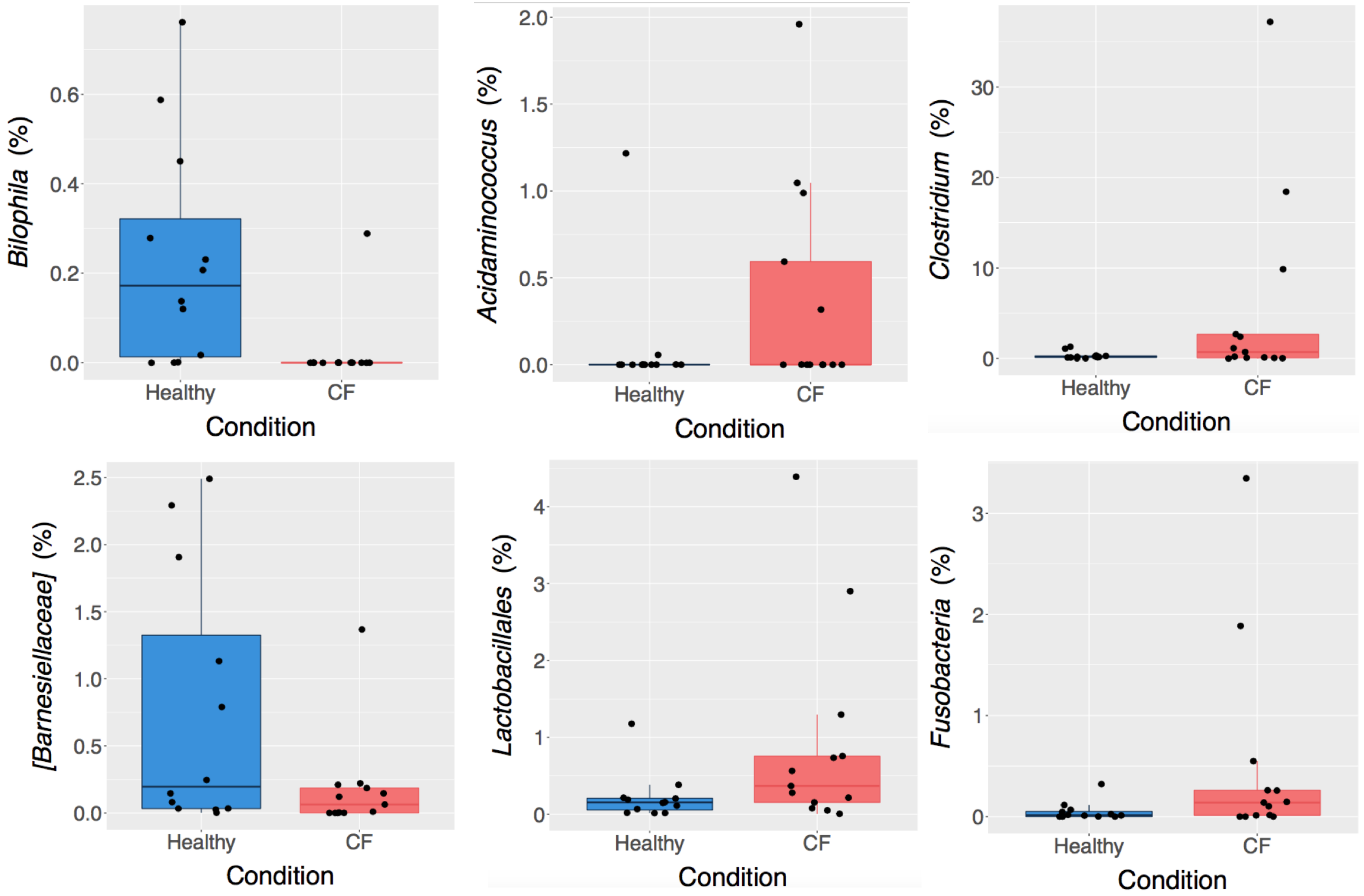

Fig S16. Randomly selected differentially abundant taxa between CF and Healthy Conditions (FDR < 0.1, Wald's test). Y-axis denotes percent relative abundance.

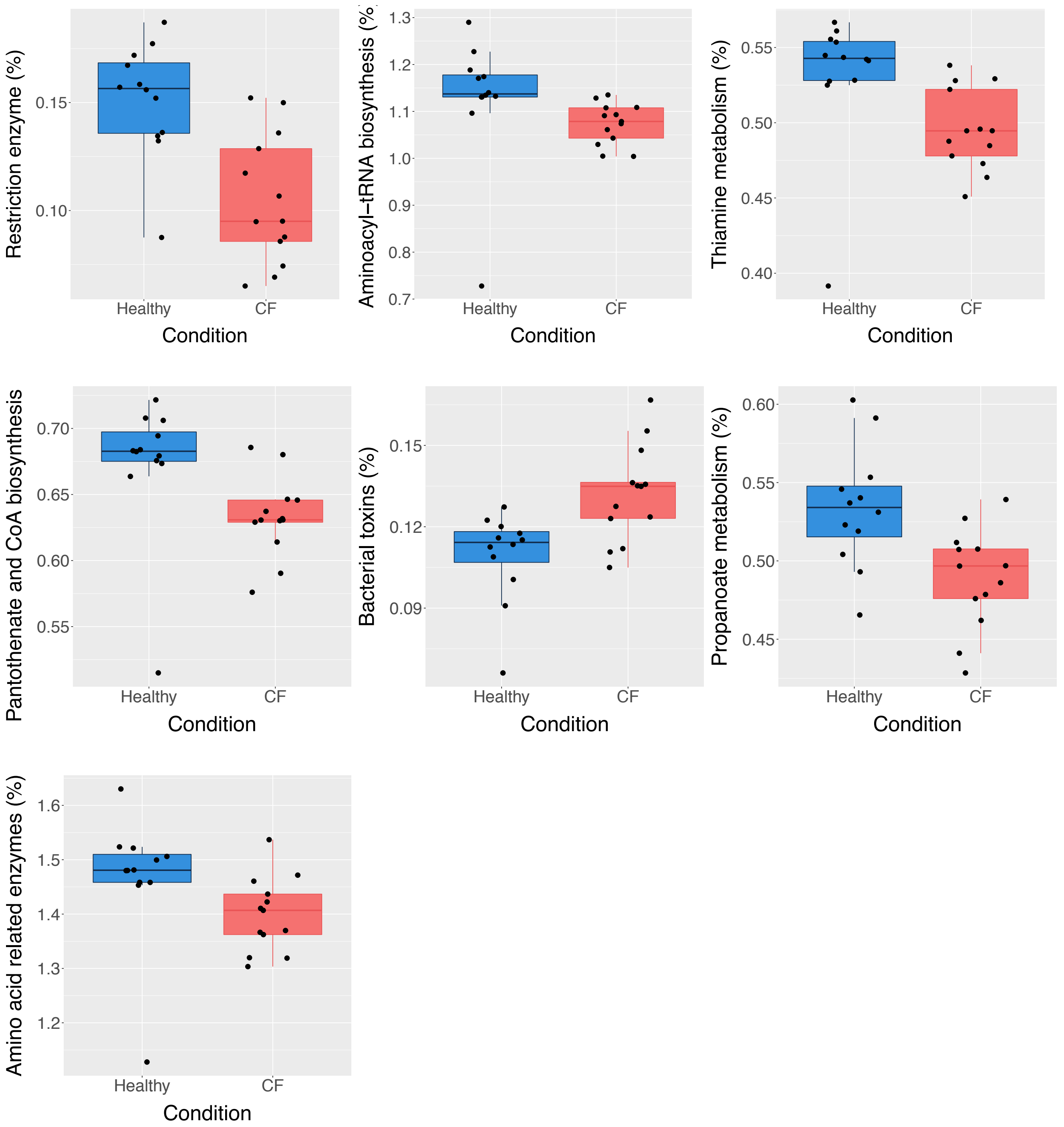

Fig S17. Differentially abundant predicted metabolic pathways in CF samples compared to healthy (FDR < 0.2, Wilcoxon rank sum test). These predicted functional profiles were generated using PICRUSt, where pathways and enzymes were assigned using KEGG database.
